# A comprehensive investigation of lipid-transfer proteins from *Cicer arietinum* disentangles their role in plant defense against *Helicoverpa armigera-infestation*

**DOI:** 10.3389/fgene.2023.1195554

**Published:** 2023-06-30

**Authors:** Harshita Saxena, Harshita Negi, Radhika Keshan, Pragya Chitkara, Shailesh Kumar, Amrita Chakraborty, Amit Roy, Indrakant K. Singh, Archana Singh

**Affiliations:** ^1^Department of Botany, Hansraj College, University of Delhi, Delhi, India; ^2^ Institute of Plant Breeding, Genetics and Genomics, University of Georgia, Griffin, GA, United States; ^3^ Department of Biological Sciences, University of South Carolina, Columbia, SC, United States; ^4^Bioinformatics Lab, National Institute of Plant Genome Research (NIPGR), New Delhi, India; ^5^ EVA 4.0 Unit, Faculty of Forestry and Wood Sciences, Czech University of Life Sciences Prague, Suchdol, Czechia; ^6^ Forest Molecular Entomology Lab, EXTEMIT-K, EVA 4.0, Faculty of Forestry and Wood Sciences, Czech University of Life Sciences Prague, Suchdol, Czechia; ^7^Department of Zoology, Deshbandhu College, University of Delhi, New Delhi, India; ^8^ Delhi School of Climate Change and Sustainability, Institution of Eminence, University of Delhi, Delhi, India

**Keywords:** lipid transfer proteins (LTPs), chickpea, *Helicoverpa armigera*, phylogenetic analysis, plant defense, herbivory

## Abstract

Lipid Transfer Proteins (LTPs) play a crucial role in synthesizing lipid barrier polymers and are involved in defense signaling during pest and pathogen attacks. Although LTPs are conserved with multifaceted roles in plants, these are not yet identified and characterized in *Cicer arietinum*. In this study, a genome-wide analysis of LTPs was executed and their physiochemical properties, biochemical function, gene structure analysis, chromosomal localization, promoter analysis, gene duplication, and evolutionary analysis were performed using *in silico* tools. Furthermore, tissue-specific expression analysis and gene expression analysis during pest attack was also conducted for the LTPs. A total of 48 LTPs were identified and named as *CaLTPs*. They were predicted to be small unstable proteins with “Glycolipid transfer protein” and “Alpha-Amylase Inhibitors, Lipid Transfer and Seed Storage” domains, that are translocated to the extracellular region. *CaLTPs* were predicted to possess 3–4 introns and were located on all the eight chromosomes of chickpea with half of the *CaLTPs* being localized on chromosomes 4, 5, and 6, and found to be closely related to LTPs of *Arabidopsis thaliana* and *Medicago trancatula*. Gene duplication and synteny analysis revealed that most of the *CaLTPs* have evolved due to tandem or segmental gene duplication and were subjected to purifying selection during evolution. The promoters of *CaLTPs* had development-related, phytohormone-responsive, and abiotic and biotic stress-related cis-acting elements. A few *CaLTP* transcripts exhibited differential expression in diverse tissue types, while others showed no/very low expression. Out of 20 jasmonate-regulated *CaLTPs*, 14 exhibited differential expression patterns during *Helicoverpa armigera*–infestation, indicating their role in plant defense response. This study identified and characterized *CaLTPs* from an important legume, *C. arietinum*, and indicated their involvement in plant defense against *H. armigera*-infestation, which can be further utilized to explore lipid signaling during plant-pest interaction and pest management.

## 1 Introduction

The persistence of environmental stresses has impelled a decline in the quality and quantity of important crops, posing severe challenges to global food and economic security. Amongst the biotic factors, insect pests, pathogens, and animals contribute to one-third of crop losses worldwide (Dhaliwal et al., 2015; Sharma et al., 2017). Plants, being sessile, are exposed to numerous such factors which are detrimental to their growth and development, and have thus, evolved various intrinsic mechanisms in response to stressful conditions. The preformed structures like spines, thorns, and trichomes can be the first level of defense employed by the plants in response to stress. Certain plant defense responses in plants are instigated by insect infestation, referred to as the induced defense mechanisms, that may be operated directly or indirectly. The indirect mode of action curtails the performance of the insects by releasing volatile compounds, such as terpenes, nitrogenous compounds, and indoles, to name a few. The direct responses include alteration in defense-associated protein expression, cell wall modifications, release of secondary metabolites and synthesis of reactive oxygen species (Kessler and Baldwin, 2002; Singh et al., 2008; Pandey et al., 2017; Alhoraibi et al., 2019). The direct defense may also include the release of phytohormones such as jasmonate (MeJA), ethylene, and salicylates (Reymond and Farmer, 1998). MeJA modulates the JA-dependent insect defense genes and plant signaling (Reyes-Díaz et al., 2016; Yu et al., 2018). Therefore, understanding the plant-insect interactions, genes/proteins that regulate the defense mechanisms, changes in their expression and their mode of action is crucial to formulate strategies for protecting crops against insect herbivory (Pandey et al., 2017).

A classic herbivory instance is the attack by the gram pod borer, *H. armigera*-infestation on *C. arietinum* L., an essential pulse and widely cultivated food legume in Asia and Africa. Being the most important pest of chickpea, *H. armigera* has led to severe losses in chickpea production by feeding straight on the foliage and pods, causing colossal damage, and eventually affecting the grain yield (Gowda et al., 2005; Sarmah et al., 2012; Rasool et al., 2015; Khatodia et al., 2017). Generally, the oral secretions of insect herbivores comprise several elicitors, that trigger the differential defense responses in plants. During the injury caused by *H. armigera* on chickpea, a plethora of changes occur at the molecular and cellular levels such as transcriptional changes and protein synthesis/degradation (Singh et al., 2008; Kerchev et al., 2012). Singh et al., 2008, have identified an array of transcripts that are differentially expressed at the time of *H. armigera* -infestation on chickpea, which contribute to plant defense against biotic and abiotic stresses, detoxification, signaling, gene regulation, and protein synthesis. In the above study, one defense-related gene, a member of LTP, was also reported to be involved in plant defense. LTPs play a crucial role in synthesizing lipid barrier polymers such as wax, suberin, and sporopollenin and are involved in defense signaling during pathogen attacks. Nevertheless, other members of the LTP family within the chickpea genome are not yet identified and their function during herbivore attacks also remains elusive.

LTPs are small, ubiquitous, lipid-binding cytosolic proteins, having a molecular mass ranging from 7 to 9 kDa and high isoelectric points, which facilitate the exchange of wide-ranging lipids between cellular membranes *in vitro*. LTP1 and LTP2 are the two major families of LTPs categorized based on their molecular weight. Since the LTPs are not directly associated with the cell walls but are found to be localized in the extracellular regions, they do not entirely suffice the role in intracellular lipid transfer, as proposed by their name. However, they are widely dispersed in the plants such as in the vascular tissues and over exposed surfaces and are encoded by multigene families in the cells (Arondel and Kader, 1990; García-Olmedo et al., 1995; Carvalho and Gomes, 2007; Yeats and Rose, 2008). Alternately, they are known to participate in various biological activities such as direct defense against pathogens as well as abiotic stresses, signaling, loosening of the cell wall and cell expansion, cutin synthesis, cuticular wax accumulation, liquid secretion, modulators of plant growth and seed, pollen, and fruit development, embryogenesis, symbiosis (Kader, 1996; Salcedo et al., 2007; Yeats and Rose, 2008; Salminen et al., 2016; Edqvist et al., 2018).

Since the discovery of LTPs in potato by J.C. Kader in 1975, it has been an abiding area for research in plant systems (Kader, 1975; Arondel and Kader, 1990; Kader, 1996). Previously, several reports give an account of LTPs identified in plants, such as 1 in *Amaranthus hypochondriacus* (del Carmen Ramírez-Medeles et al., 2003), 49 in *Arabidopsis thaliana* (Boutrot et al., 2008), 14 in *Artemisia annua* (Adhikari et al., 2019), 13 in *Astragalus sinicus* (Chou et al., 2006), 1 in *Beta vulgaris* (Kristensen et al., 2000)*,* 39 in *Cucumis sativus* (Wang et al., 2020), 40 in *Hordeum vulgare* (Duo et al., 2021), 52 in *Oryza sativa* (Boutrot et al., 2008), 2 in *Ricinus communis* (Takishima et al., 1988)*,* 5 in *Sesamum indicum* (Choi et al., 2008) and 83 in *Solanum tuberosum* (Li et al., 2019)*.* Although LTP family members had been identified and characterized in model and a few crop plants, studies on leguminous crop plants including chickpea have not been explored much.

Chickpea is the third most crucial leguminous crop and their production is severely affected by many biotic and abiotic stresses. Amongst the biotic stresses, *H. armigera*-infestation causes substantial crop losses (Singh et al., 2008; Kumar et al., 2020). *C. arietinum*-*H. armigera* interaction has been investigated at the molecular level (Sanyal et al., 2005; Jaiswal et al., 2012; Dinesh et al., 2017; Singh et al., 2017; Singh et al., 2018a; Singh et al., 2018b; Singh et al., 2018b; Singh et al., 2018c; Barmukh et al., 2021), but the role of LTPs in chickpea plant defense against *H. armigera* has not been attempted yet. Therefore, we identified members of LTPs in the chickpea genome and characterized them. Through this study, the roles of LTPs in the Fabaceae (Leguminosae) family will be further enriched, as we have identified 48 LTPs from *C. arietinum* (hereby named CaLTPs). In addition to evaluating their physiochemical properties, chromosomal and subcellular localization, we have also investigated their distribution patterns of introns and exons, motif analysis, and phylogenetic and evolutionary relationships. We have also used RNA-seq data to evaluate the expression of *CaLTPs* in different tissues and performed relative expression analysis to identify the *CaLTPs* that are potentially involved in defense against *H. armigera* -infestation. Our results provide an extensive analysis of CaLTPs and contribute information on the role of CaLTPs in plant defense.

## 2 Materials and methods

### 2.1 Identification, characterization, and localization of CaLTPs

The Hidden Markov Model profiles of the LTP domain were taken (http://Pfam.sanger.ac.uk/) and searched against chickpea genome to detect the LTP proteins in the *C. arietinum* with the E value <0.05. Sequences of all hits were retrieved and redundant sequences were removed and the selected sequences were scanned through HMMSCAN (http://hmmer.janelia.org/search/hmmscan) to check for the presence of conserved LTP domain. For further confirmation, selected sequences were utilized to check their similarity with *A. thaliana* LTPs using BLASTp and tBLASTn with an identity of 50% as the threshold. The genomic and peptide sequences of the CaLTPs were checked manually at NCBI, using both BLASTn and BLASTp, for further confirmation. The physicochemical properties, like isoelectric point (pI), molecular weight, number of amino acids, negatively and positively charged residues, instability, aliphatic indices, and grand average of hydropathicity (GRAVY), were enumerated for the candidate CaLTPs, using the ExPASy ProtParam (https://web.expasy.org/protparam/) tool (Walker, 2005). The localization in the subcellular compartments was interpreted using the web tool, Wolf PSORT (https://www.genscript.com/wolf-psort.html), which predicts the localization based on the sorting signal motif and uses the k-nearest neighbor clustering method to group the corelative sequences (Horton et al., 2007)

### 2.2 Chromosomal localization and promoter analysis of *CaLTPs*

The ‘Genome’ extension (https://www.ncbi.nlm.nih.gov/genome/?term=) of the NCBI database was used to discern the chromosomal lengths of the 8 chickpea chromosomes (Bethesda, 1988). The chromosomal location for all the putative *LTPs* from chickpea, retrieved from NCBI earlier, was used to mark the positions of the candidate *LTPs* on the chickpea chromosomes, using the MapChart (v2.32) software, downloaded from MapChart (https://www.wur.nl/en/show/mapchart.htm) (Voorrips, R.E., 2002). The promoter sequences (1 kb genomic sequences present upstream to the transcription initiation site) of the *CaLTPs* were retrieved from the NCBI database and the analysis for the presence of different motifs in these 1,000 bp sequences was performed using the database, PlantCARE (http://bioinformatics.psb.ugent.be/webtools/plantcare/html/) (Lescot et al., 2002).

### 2.3 Gene structure and motif analysis of CaLTPs

The structural organization of introns and exons in the candidate genes was analyzed and visualized using the Gene Structure Display Server v2.0 (http://gsds.gao-lab.org/) (Hu et al., 2015). The presence of novel conserved motifs in the LTP gene family of chickpea was identified using the MEME suite tool v.5.3.3 (https://meme-suite.org/meme/), such that the number of characters in a sequence pattern defined as the maximum optimal width was selected 200, with a motif limit of 10 (Bailey and Elkan, 1994; McLeay and Bailey, 2010; Bailey et al., 2015). The functions of the identified motifs were retrieved from the Conserved Domain Database (CDD) (https://www.ncbi.nlm.nih.gov/Structure/cdd/wrpsb.cgi) (Marchler-Bauer et al., 2015).

### 2.4 Multiple sequence alignment and phylogenetic analysis of LTPs

The evolutionary relatedness of the LTP protein family of chickpea with the LTPs characterized in *A. thaliana, O. sativa,* and *M. truncatula* was analyzed using the Molecular Evolutionary Genetic Analysis version X (MEGA X) (https://www.megasoftware.net/) software (Kumar et al., 2018). The LTP sequences from the reference organisms, *A. thaliana, O. sativa,* and *M. truncatula*, were retrieved from the UniProt database (https://www.uniprot.org/) (The UniProt Consortium, 2021). The multiple sequence alignment (MSA) of all the sequences was performed by the ClustalW method in MEGA X. Maximum Likelihood (ML) method of tree construction was employed to construct the evolutionary tree, with 1,000 bootstraps per replication, which was visualized using an online tool, Interactive Tree Of Life (iTOL) v6.3 (https://itol.embl.de/) (Letunic and Bork, 2021).

### 2.5 Gene duplication and ka/ks value calculation of *LTPs*

The Multiple Collinearity Scan Toolkit (MCScanX) was used to analyse the homologous gene pairs in the identified *CaLTP*s within the *C. arietinum* genome, as well as their orthologous relationship with the genes of *A. thaliana, O. sativa,* and *M. truncatula* that have evolved due to duplication (Wang et al., 2012). The top five hits of an all-versus-all local BLAST search for the protein sequences of the aforementioned species were utilised as input in MCScanX to acquire data on collinear pairs of genes. The CIRCOS software package was also utilised to map the identified duplicated gene pairs using the collinearity file obtained (Krzywinski et al., 2009). The selection pressure of the *CaLTPs* was evaluated by finding the ratio of synonymous (Ks) and nonsynonymous (Ka) substitutions for each duplication using the PAL2NAL web server (http://www.bork.embl.de/pal2nal/) (Suyama et al., 2006). A pairwise alignment result for each duplicated gene pair and their CDS sequences was used as an input in the software.

### 2.6 Gene-ontology-based functional annotation of CaLTPs

The functional characterization of the LTP gene family of chickpea was performed using the functional analysis module of OmicsBox https://www.biobam.com/omicsbox/). Blast2GO, InterPro, and EggNOG plugins were used to perform gene-ontology-based functional characterization of the LTP gene family of chickpea (Conesa et al., 2005; Conesa and Götz, 2008).

### 2.7 Identification of microRNA (miRNA) targets of *CaLTPs*

An online plant small RNA analysis server, psRNATarget was used to predict possible miRNA sequences that can bind with the *CaLTPs* (Dai et al., 2018). The CDS sequence of the *CaLTPs* was used as the target and scoring schema V1 was used to perform the analysis with expectation value set at 3 and other default parameters.

### 2.8 Growth of chickpea and maintenance of *H. armigera culture*

The methodology employed by Armes et al. (1992), Singh et al. (2008), Singh et al. (2017), and Keshan et al. (2021), for the growth of chickpea plants and the *H. armigera* culture, were used for this study as well (Armes et al., 1992; Singh et al., 2008; Singh et al., 2017; Keshan et al., 2021). Briefly, the chickpea seeds (Pusa 362) were washed and soaked overnight. The soaked seeds were transferred to the sterilized soilrite containing pots. The seeds were allowed to germinate and seedlings were grown in a plant growth chamber under controlled conditions (photoperiod: 16 h light and 8 h dark; temperature: 26–27°C; humidity: 55%–60%) and watered regularly. For rearing and maintenance of *H. armigera,* the larvae were procured from ICAR–National Bureau Of Agricultural Insect Resources, Bangalore, India, and reared in the laboratory on chickpea -fluor based artificial diet using standard protocols at 27°C and 65%–70% relative humidity on a 14/10 h light/dark cycle (Armes et al., 1992; Singh et al., 2008). The freshly molted fifth instar larvae (one of the most active and infesting stage) were starved for 12 h before releasing them on plants for experimentation and bioassays.

For infestation, the fifth-instar larvae were allowed to starve overnight and exposed to one-month-old chickpea plants (one larva per plant). The infestation by the larvae was allowed until 20% of the infestation was achieved. Plants without any treatments served as control. The whole shoot of treated and control samples were collected, snap-frozen in liquid nitrogen, and stored at −80°C.

### 2.9 Isolation of total RNA and cDNA synthesis

Total RNA was isolated from the leaf tissues using TRIzol^TM^ Reagent (Invitrogen, USA), as per the instructions from the manufacturer, and the quality and quantity of the RNA were checked as per previously published articles (Singh et al., 2017; Singh et al., 2018b). The cDNA was synthesized using 1 μg of RNA using the cDNA synthesis kit (script select cDNA synthesis kit, Bio-rad).

### 2.10 Relative gene expression of MeJA-responsive *CaLTPs*

From the promoter analysis of the putative chickpea *LTPs*, 21 *LTPs* were found to have motifs for MeJA responsiveness. Primers were designed for these 20 *CaLTPs* (Supplementary Table S6) having MeJA responsiveness as well as for the internal control genes (actin), using the web tool, Primer3 v. 0.4.0 (https://bioinfo.ut.ee/primer3-0.4.0/), with conditions such as primer size, primer T_m_, primer GC%, and product size ranging from 18–23 bp, 60°C–62°C, 40%–70%, and 150–200 bp, respectively, and the maximum poly-X as 3 (Koressaar and Remm, 2007; Untergasser et al., 2012; Kõressaar et al., 2018). The quantitative real-time PCR (qRT-PCR) was performed to check the relative expression of the *CaLTPs* in reference to the internal control genes using the SYBR Green Master Mix (Biorad) on ABI step one Real-time (Applied Biosystems). The real-time data obtained was then used for determining ∆C_t_, ∆∆C_t_, and RQ values. The relative expression values were statistically analyzed using ANOVA and Student’s t-test.

### 2.11 Tissue-specific expression analysis

To access the tissue-specific expression, Chickpea Transcriptome Database (CTDB) (http://www.nipgr.res.in/ctdb.html) was used to retrieve the identifiers for each of the 48 *CaLTPs* and RNAseq data corresponding to the differential expression in root, shoot, flower bud, mature leaf, and young pod was retrieved for each of the *CaLTP* [Reads/Kb/Million (RPKM) normalized data] (Verma et al., 2015). The heatmap.2 function from the ggplot2 package of RStudio was used to generate the heatmap (R Studio, 2020).

## 3 Results

### 3.1 Identification, characterization, and localization of CaLTPs

**FIGURE 1:**
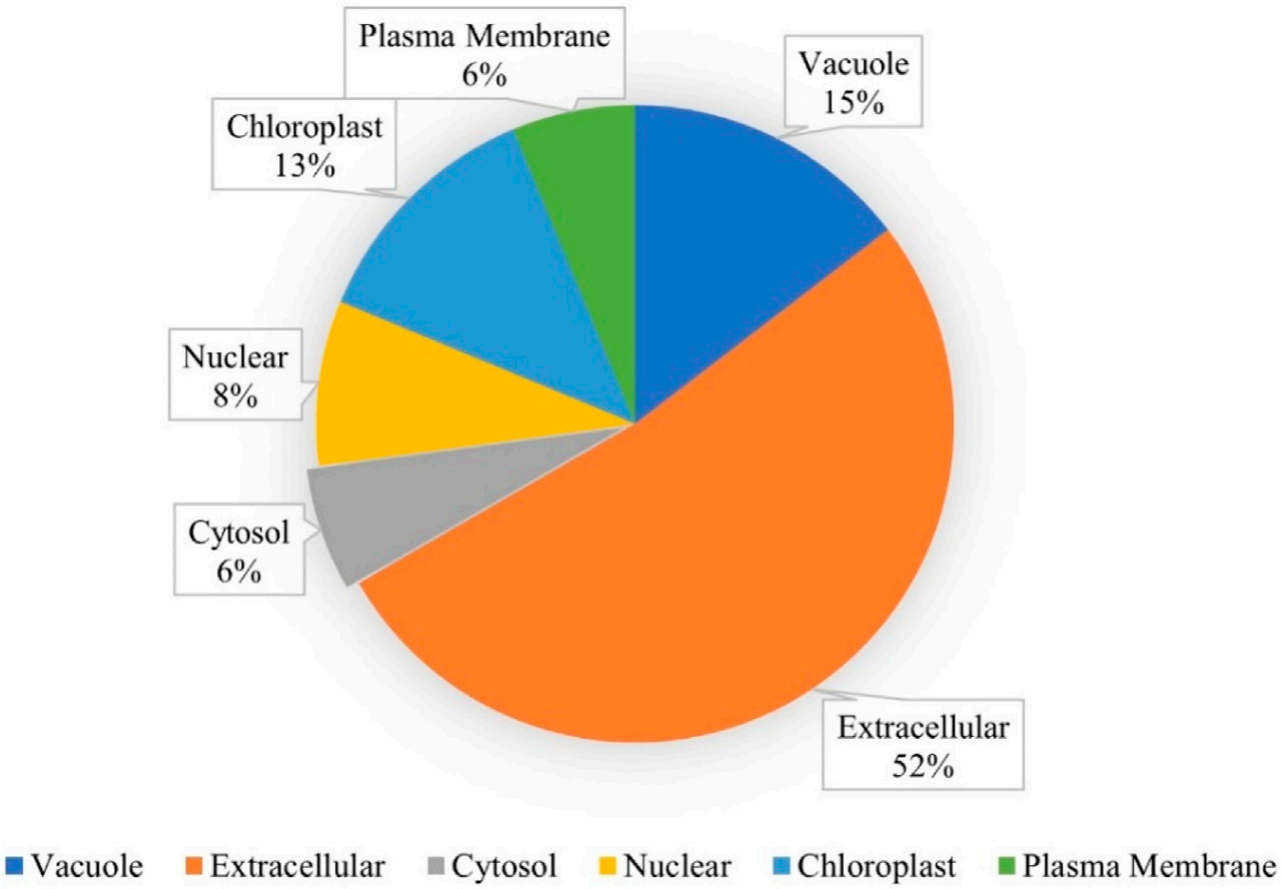
Subcellular localization of the CaLTPs using WolfPSORT, showing 52, 15, 13, 8, 6, and 6 percent of CaLTPs localized in the extracellular membranes, vacuole, chloroplast, nuclear, cytosol, and plasma membranes, respectively.

A total of 48 lipid transfer proteins (LTPs) were identified in the chickpea genome using *in silico* analysis (HMM profiles, HMMER scan and homology search; Supplementary Figures S1, S2). These 48 LTPs were given appropriate nomenclature from CaLTP1 to CaLTP48 (Supplementary Table S1), based on their location on the chickpea chromosomes, deciphered using the National Center for Biotechnology Information (NCBI) (https://www.ncbi.nlm.nih.gov/) (Bethesda, 1988) (Table 1). Evaluation of their physiochemical properties revealed that the number of amino acids ranged from 92 to 256 with the molecular weight ranging from 9971.93 to 29308.41 Da. Their pI varied from 3.9 to 9.91 and 31 out of 48 proteins were predicted as unstable because their instability index was greater than 40. The aliphatic index of side chains varied from 78.51 to 136.59 and the GRAVY values indicated the hydrophilicity of the proteins as the values were found to be <0. Sub-cellular localization performed via WolfPSORT indicated that the proteins majorly translocated in the extracellular region which suggested their roles in lipid transport between membrane bilayers (Table 1; Figure 1)

**TABLE 1:** Physicochemical properties (pI, molecular weight, number of amino acids, number of negatively and positively charged residues, net charge, instability and aliphatic indices, GRAVY) and subcellular localization of the 48 CaLTPs.

S.No.	CaLTPs	pI	Mol. Wt.	Amino acids	Negatively charged residues	Positively charged residues	Net charge	Instability index	Aliphatic index	Grand average of hydropathicity (GRAVY)	Sub-cellular localization	
											Prediction by WoLF PSORT	No. of nearest neighbour to the query
1	CaLTP1	7.48	18160.05	181	8	9	1	52.87 (Unstable)	95.52	0.579	Vacuole	6
2	CaLTP2	8.61	13748.65	132	5	9	4	31.15	136.59	0.796	Extracellular	8
3	CaLTP3	6.91	22857.99	206	21	21	0	28.46	90.97	−0.117	Cytosol	8
4	CaLTP4	8.61	29308.41	256	29	32	3	36.43	89.14	−0.309	Nuclear	5
5	CaLTP5	6.16	12633.59	112	12	10	−2	33.34	88.84	0.057	Extracellular	11
6	CaLTP6	8.66	11658.55	115	7	11	4	27.89	93.39	0.257	Extracellular	11
7	CaLTP7	8.86	10919.98	104	7	12	5	39.76	91.06	0.178	Extracellular	11
8	CaLTP8	8.57	13246.50	120	10	14	4	51.17 (Unstable)	87.83	0.105	Extracellular	11
9	CaLTP9	5.27	11379.60	105	11	10	−1	42.37 (Unstable)	90.10	0.126	Extracellular	11
10	CaLTP10	8.08	12046.03	116	8	10	2	42.36 (Unstable)	83.28	0.239	Extracellular	10
11	CaLTP11	9.17	12654.10	122	1	10	9	49.35 (Unstable)	95.82	0.407	Extracellular	11
12	CaLTP12	8.73	12790.99	122	4	9	5	41.71 (Unstable)	80.74	0.228	Extracellular	8
13	CaLTP13	7.48	18162.84	174	6	7	1	55.37 (Unstable)	93.05	0.393	Vacuole	5
14	CaLTP14	8.06	18411.07	181	4	6	2	55.51 (Unstable)	79.12	0.336	Chloroplast	5
15	CaLTP15	6.89	22586.08	202	23	23	0	57.62 (Unstable)	81.14	−0.100	Nuclear	5
16	CaLTP16	3.90	11699.49	113	6	2	−4	44.09 (Unstable)	97.52	0.563	Extracellular	10
17	CaLTP17	5.98	10067.82	97	5	5	0	39.97	98.56	0.492	Extracellular	11
18	CaLTP18	8.41	22666.11	227	4	7	3	70.15 (Unstable)	85.07	0.361	Vacuole	9
19	CaLTP19	5.64	24451.10	217	28	24	−4	38.46	99.77	−0.045	Cytosol	8
20	CaLTP20	6.65	22795.23	202	23	22	−1	55.97 (Unstable)	84.50	−0.158	Nuclear	10
21	CaLTP21	6.60	25398.82	221	31	30	−1	54.33 (Unstable)	107.10	−0.151	Cytosol	5
22	CaLTP22	9.10	13056.61	126	4	12	8	36.27	113.10	0.546	Extracellular	11
23	CaLTP23	8.70	12293.48	114	6	11	5	33.21	93.25	0.230	Extracellular	11
24	CaLTP24	9.00	14706.52	142	5	12	7	53.65 (Unstable)	96.2	0.293	Extracellular	12
25	CaLTP25	8.56	14634.31	145	6	10	4	57.44 (Unstable)	102.28	0.378	Vacuole	6
26	CaLTP26	4.96	20681.74	209	14	11	−3	59.12 (Unstable)	89.23	0.365	Vacuole	9
27	CaLTP27	6.11	19562.75	184	19	18	−1	47.37 (Unstable)	108.04	0.241	Plasma membrane	6
28	CaLTP28	5.82	19575.94	185	17	16	−1	45.34 (Unstable)	94.38	0.151	Vacuole	8
29	CaLTP29	7.77	25081.94	220	29	30	1	51.11 (Unstable)	96.59	−0.33	Nuclear	6
30	CaLTP30	8.57	21046.49	194	16	20	4	44.49 (Unstable)	95.46	0.025	Extracellular	7
31	CaLTP31	7.48	20808.2	193	18	19	1	34.5	97.98	0.101	Extracellular	7
32	CaLTP32	4.87	18446.15	188	10	7	−3	51.08 (Unstable)	91.38	0.536	Vacuole	11
33	CaLTP33	8.92	11687.77	116	3	9	6	26.26	95.17	0.505	Extracellular	13
34	CaLTP34	9.91	12672.05	119	3	17	14	39.08	80.42	0.1	Extracellular	13
35	CaLTP35	8.75	13064.03	124	3	8	5	34.43	83.31	0.219	Extracellular	7
36	CaLTP36	6.68	11512.52	105	9	9	0	35.43	85.43	0.15	Chloroplast	6
37	CaLTP37	9.85	12762.4	122	5	18	13	43.29 (Unstable)	103.28	0.406	Extracellular	9
38	CaLTP38	5.88	20834.1	196	18	16	−2	40.9 (Unstable)	87.6	0.012	Extracellular	7
39	CaLTP39	7.48	21348.56	210	7	8	1	69.4 (Unstable)	84.05	0.254	Plasma membrane	5
40	CaLTP40	8.64	17043.4	172	3	7	4	59.64 (Unstable)	80.06	0.191	Chloroplast	9
41	CaLTP41	9.22	9971.93	92	2	10	8	47.77 (Unstable)	89.02	0.201	Extracellular	8
42	CaLTP42	9.17	11722.75	118	2	10	8	43.28 (Unstable)	80.34	0.468	Extracellular	14
43	CaLTP43	8.4	16173.81	154	10	13	3	61.07 (Unstable)	78.51	0.098	Plasma membrane	5
44	CaLTP44	8.91	13871.64	131	5	11	6	35.49	107.18	0.418	Extracellular	9
45	CaLTP45	9.16	16260.14	157	4	13	9	44.33 (Unstable)	90.13	0.043	Extracellular	7
46	CaLTP46	6.5	14321.82	135	6	6	0	54.05 (Unstable)	101.85	0.43	Chloroplast	4
47	CaLTP47	8.89	16840.47	166	4	10	6	63.03 (Unstable)	88.07	0.16	Chloroplast	11
48	CaLTP48	7.49	11602.78	107	5	6	1	37.69	97.48	0.616	Chloroplast	6

### 3.2 Chromosomal localization and promoter analysis

**FIGURE 2:**
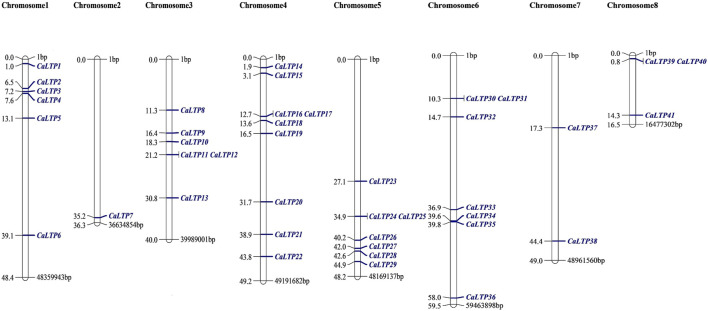
Mapping of the 48 CaLTPs on the eight chickpea chromosomes using MapChart software. The CaLTPs are depicted using blue font (right side of each chromosome), and their exact position on the chromosome (in base pairs, bp) is mentioned next to each LTP (left side of each chromosome).

**FIGURE 3:**
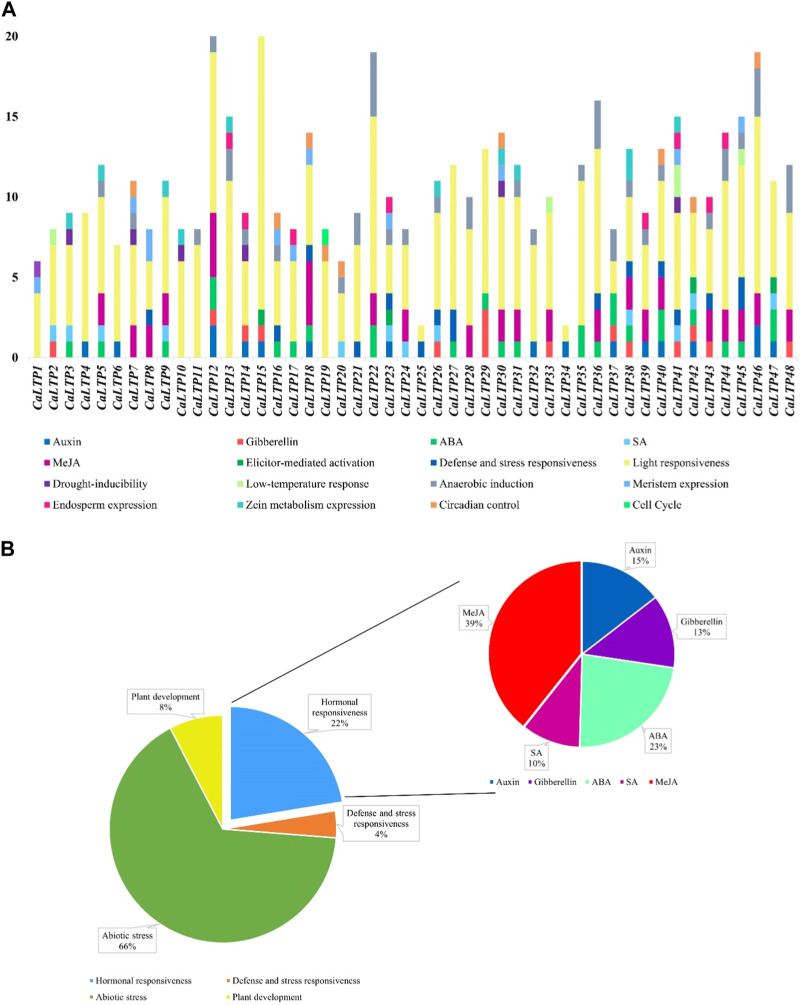
Promoter analysis of the CaLTPs. **(A)** Different promoter elements in CaLTPs and their role in different responses; **(B)** Pie chart depicting some LTPs participating in external responses and plant developmental process, emphasizing the roles in hormonal responsiveness.

The 48 *CaLTPs* were seen to be localized on all eight chromosomes (haploid) of *C. arietinum* with the highest number of *LTPs* located on chromosome 4 (Figure 2). Most of the genes were present on the distal end of the chromosome suggesting their frequent involvement in the recombination process and might have high recombination frequency, indicating the potential causes of functional divergence of *CaLTPs.* The genes allocated at the proximal end of the chromosomes are highly unlikely to undergo recombination. Promoter analysis identified many cis-acting elements having roles in hormonal responsiveness, defense and stress responsiveness, abiotic stress, and plant development. Most of the *CaLTPs* transcription elements (about 66%) had roles in abiotic stress responses such as light responsiveness, drought-inducibility, low-temperature response, and anaerobic induction (Figure 3A; Supplementary Figure S3; Supplementary Table S2). Very few (∼8%) *CaLTPs* had promoter elements that contributed toward plant development. About 22% of the promoter elements showed hormonal responsiveness towards auxin, gibberellin, ABA, SA, and MeJA. It was also interesting to note that out of all the *CaLTPs,* 20 *CaLTPs*’ promoter sequences had elements responsive towards MeJA (Figure 3B). This is suggestive of the participation of *CaLTPs* in defense responses against herbivory and insect attacks by initiating the MeJA-induced defense mechanism.

### 3.3 Structural analysis and motif arrangement

**FIGURE 4:**
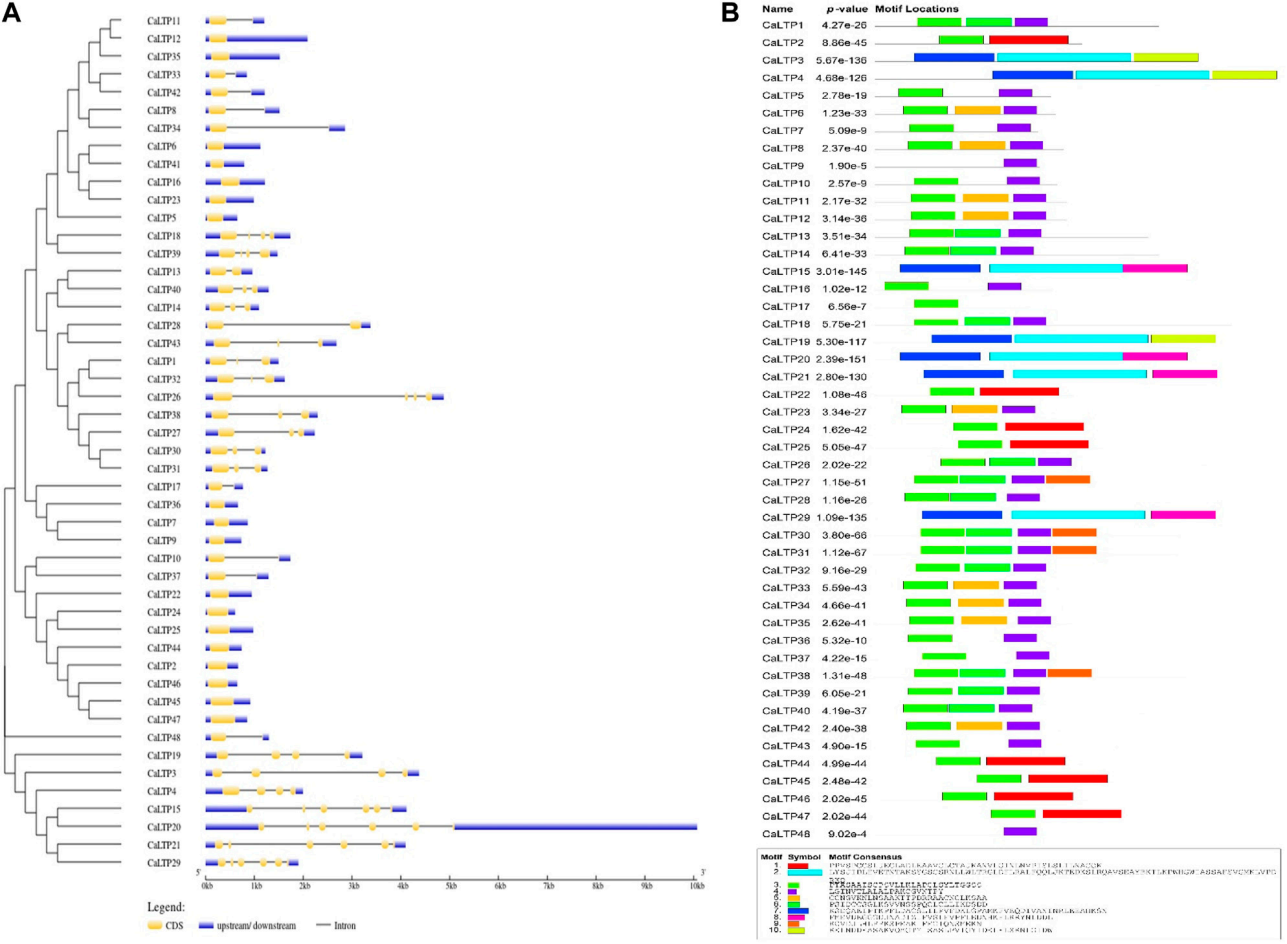
Gene structure and motif analyses. **(A)** The distribution patterns of exons and introns in the LTP gene family of chickpea were assessed using the GSDS server. Blue, yellow, and black colors, respectively represent the upstream/downstream, exons and introns **(B)** The presence of 10 novel conserved motifs in the CaLTPs was analyzed using the MEME suite.

The distribution pattern of the exons and introns and their positions in the respective genes were analyzed using the GSDS server (Figure 4A). The maximum number of exons was found in *CaLTP 12* and *29*. No introns were found in 18 out of 48 *CaLTP*s. More than half of the *LTPs* had three or fewer introns and with 5 introns as the maximum number. This array of exons and introns helps to stipulate the structural, functional, and evolutionary features of the genes. Furthermore, the MEME suite helped to discover the ten novel conserved motifs for the 48 CaLTP genes (Figure 4B). The identified motifs were associated with the AAI_LTSS and GLTP superfamily and showed major involvement in the lipid transfer activity. The width of the novel motifs ranged from 21 to 85 amino acids. Motifs 3 and 4 were present in almost all the 48 *CaLTPs* and may correspond to the core LTP domain responsible for intermembrane lipid transfer activity. Motif 10 was present only in CaLTP 3, 4, and 19 while motifs 9 and 8 were found in CaLTP 30, 31, 27, 38, and CaLTP 15, 20, 29, and 21, respectively, suggesting that these three motifs might have some role exclusive to the protein they are present in. It was interesting to note that Motif 7 was only present in the CaLTPs (CaLTP 3,4,15,19,20,21, and 29), which had either motif 10 or motif 8 suggesting a hallmark linkage between the motifs (Supplementary Figure S4; Supplementary Table S3).

### 3.4 Phylogenetic relatedness amongst CaLTPs and LTPs from model plants

**FIGURE 5:**
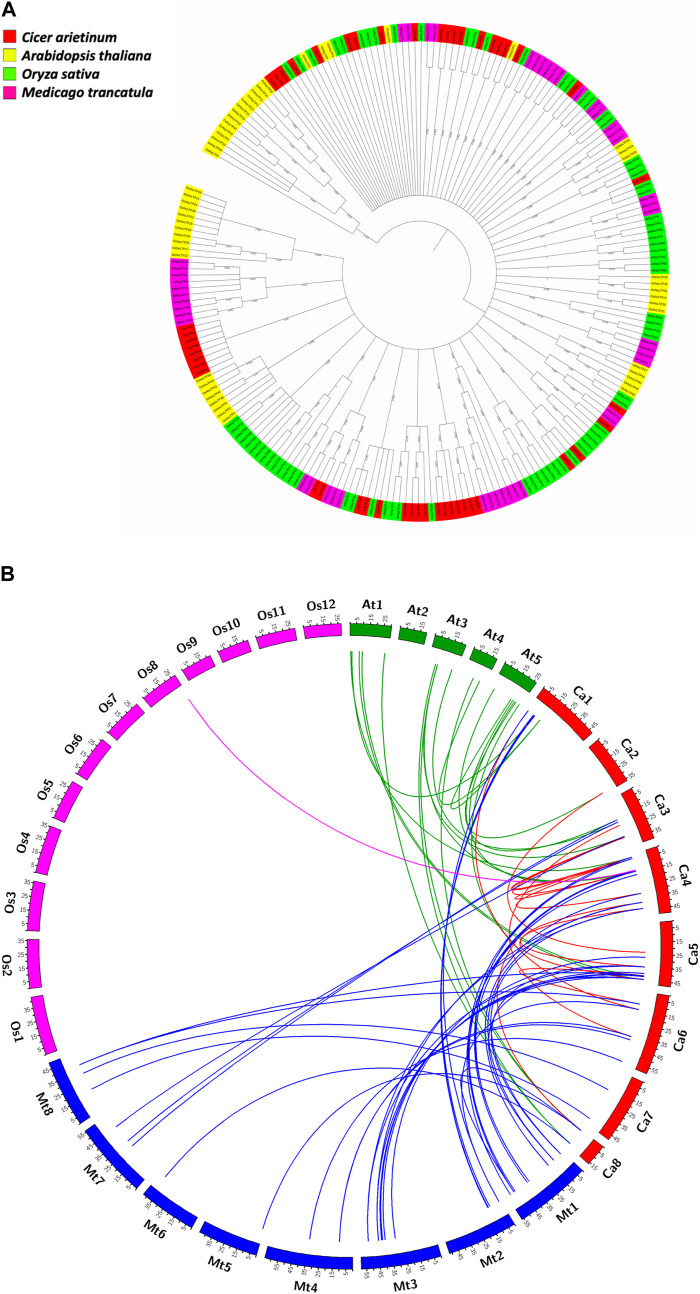
Evolutionary studies of CaLTPs. **(A)** The phylogenetic tree depicting the evolutionary relatedness of the LTP gene family in chickpea to those in the reference plant systems like *Arabidopsis thaliana*, *Oryza sativa*, and *Medicago truncatula*. **(B)** Segmentally duplicated LTP pairs between *C. arietinum* (red) and *Arabidopsis thaliana* (green); *C. arietinum* (red) and *Medicago trancatula* (blue); *C. arietinum* (red) and *Oryza sativa* (pink).

The evolutionary relationships of the LTP gene family in *C. arietinum* were studied with those in model dicots *A. thaliana* and *M. truncatula,* and model monocot *O. sativa,* by constructing a phylogenetic tree (Figure 5A). The results indicate that the LTPs have evolved significantly over a period of time, which is evident from different clades in the tree, suggesting the diverse roles of LTPs in the different plant systems. Out of 48 CaLTPs, 18 LTPs were found to occur in two clades next to each other, depicting relatedness amongst each other and no signs of functional divergence or relatedness with LTPs from reference organisms. The remaining LTPs were dispersed throughout the phylogenetic tree and were part of different clades sharing evolutionary relationships with the LTPs from *A. thaliana* and *M. truncatula.* The LTPs from *O. sativa* were present in distinct clades showing no signs of evolutionary relatedness with the chickpea LTPs.

### 3.5 Occurrence of gene duplication in *LTPs* and ka/ks value calculation

Gene duplication events play an essential role in expanding gene families leading to species divergence and evolving novel gene functions through subfunctionalization and neofunctionalization (Innan and Kondrashov, 2010). Analyzing the occurrence of gene duplication events within the *C. arietinum* genomes showed that a total of 16 pairs of genes underwent duplication, with four tandem duplication events, where the duplicated pairs were found on the same chromosome; 12 segmental duplication events, where the duplicated genes were present on different chromosomes (Supplementary Table S1B). Interestingly, all the tandemly duplicated gene pairs were present on chromosome 4. A comparative genome mapping of *CaLTPs* with genes from *A. thaliana, O. sativa,* and *M. truncatula* was also analyzed to show an orthologous relationship of the *CaLTP*s with genes from these species (Figure 5B). A total of 67 pairs of orthologous duplicated genes were identified, including 23 pairs between *CaLTPs* and the genes of *A. thaliana*, 43 pairs between *CaLTPs* and the genes of *M. truncatula,* and one pair between *CaLTPs* and *O. sativa*. The *CaLTPs* showed a maximum synteny with *M. truncatula* genes (34 *CaLTPs*), followed by *A. thaliana* (18 genes). In contrast, only one gene, *CaLTP*17, was found to have an orthologous relationship with genes from *O. sativa*. The results indicate that most of the *CaLTPs* have evolved due to gene duplication events, but a few might have been present earlier. To determine the selection pressure involved in the duplication and divergence of *LTPs*, the non-homologous (Ka) and synonymous (Ks) substitution rates of duplicated gene pairs (paralogous as well as orthologous gene pairs) were determined. Within the *C. arietinum* genome, the ratio of Ka/Ks for segmentally duplicated gene pairs ranged from 0.0934 to 0.4396 (with an average of 0.21), whereas tandemly duplicated genes had an average range of 0.086. In all paralogous duplicated gene pairs, the Ka/Ks ratio varied from 0.0064 to 0.817, with an average of 0.25. All homologous pairs of genes were found to have a Ka/Ks ratio smaller than 1, indicating that the duplicated genes were subjected to purifying selection during evolution (Kondrashov et al., 2002).

### 3.6 GO-based functional annotation of CaLTPs

**FIGURE 6:**
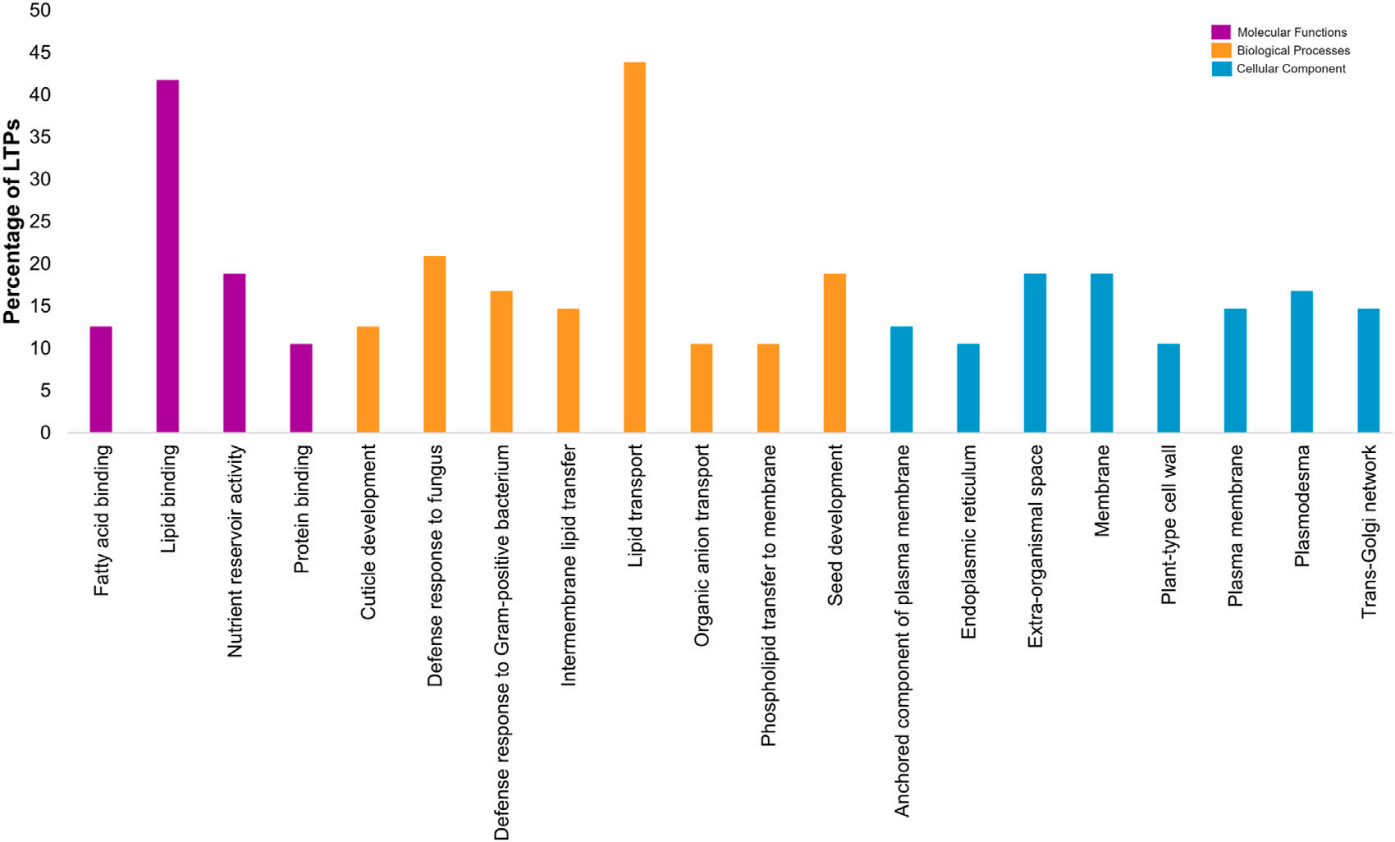
Gene-ontology-based functional annotation of the LTP gene family of chickpea, with diverse roles in Molecular functions, Biological processes and Cellular component.

Gene ontology-based functional annotation uncovered the roles of CaLTPs in various components like molecular functions, biological processes, and cellular components. A majority of *CaLTPs* took part in biological processes and molecular functions related to lipid transport (GO:0006869) and lipid binding (GO:0008289), respectively. It was interesting to note that apart from lipid transport, CaLTP genes also participated in the biological processes such as defense response to fungus (GO:0050832), seed development (GO:0048316) and defense response to Gram-positive bacteria (GO:0050830) to name a few. They also took part in molecular functions such as nutrient reservoir activity (GO:0045735), fatty acid binding (GO:0005504), protein binding (GO:0005504). The cellular component occupied by most of the LTPs were membrane (GO:0005886), extra-organismal space (GO:0043245), and plasmodesmata (GO:0009506) (Figure 6; Supplementary Table S5).

### 3.7 miRNA targets prediction for *CaLTPs*

With the default parameters and expectation value of ≤3, psRNATarget could predict 51 miRNAs that can potentially bind with the *CaLTPs* (Supplementary Table S7). The search was optimized with the expectation value (measure of similarity and the possibility for the mismatches between mature small RNA and the target sequence) and UPE (maximum energy to unpair the target site) value of 2 and 19.377 respectively.

### 3.8 Effect of *H.armigera-*infestation on the expression profiles of *CaLTPs*

**FIGURE 7:**
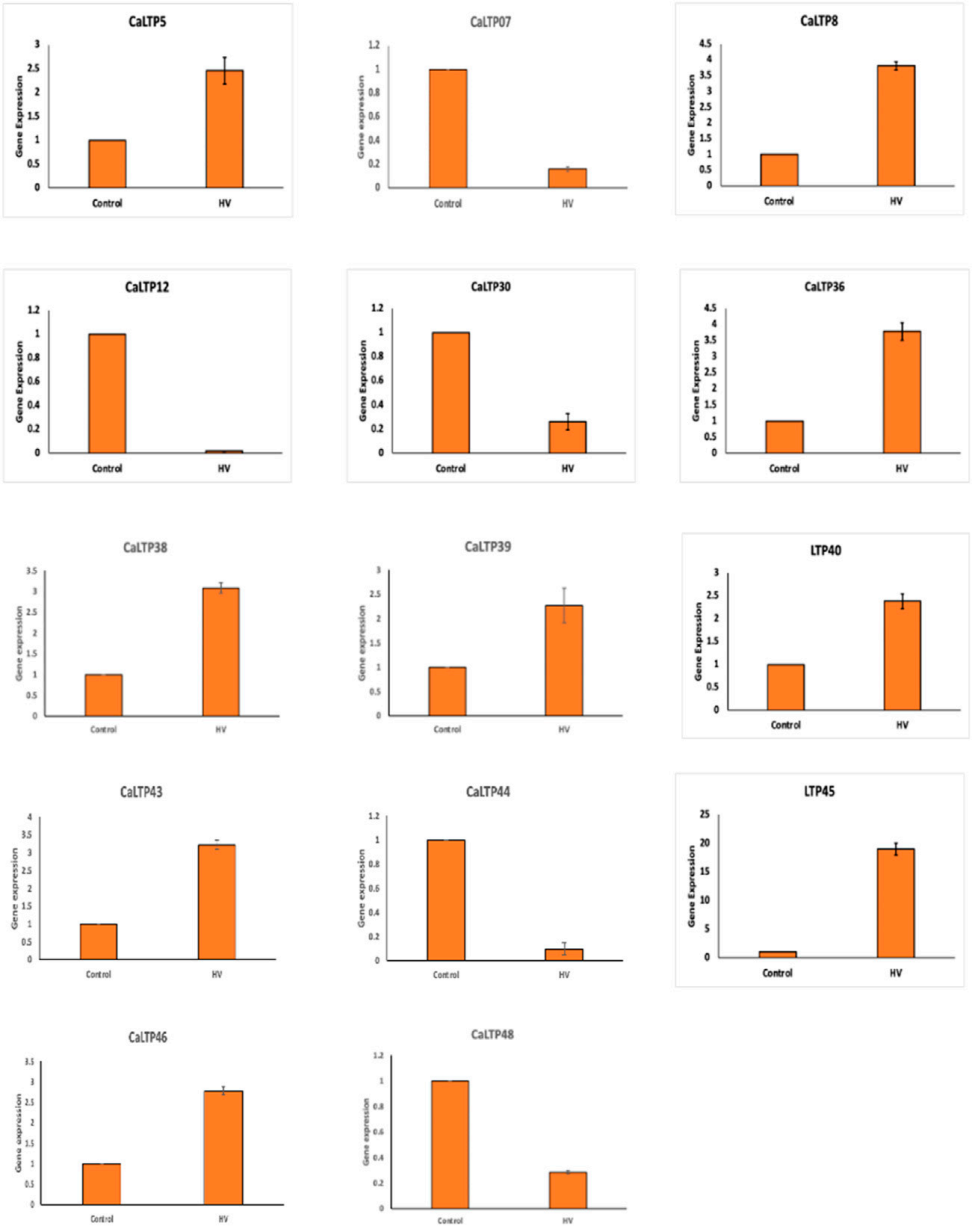
The qRT-PCR expression pattern of *CaLTPs* during *H. armigera* -infestation. Actin was used as the internal control gene. Triplicate set of data were used for each experiment. The statistical analysis was conducted using AVOVA and Student’s T-tests. The data are plotted as means ± s.d. The error bars represent standard deviations.

A total of 20 *CaLTPs* that showed MeJA responsiveness, were used for studying the relative gene expression when infested with *H. armigera.* Specific primers were designed to study the real-time expression of the 20 *CaLTPs*, where actin was used as the internal control (Figure 7). A positive fold-change was observed for 9 of the *CaLTPs* (*CaLTP 5, 8, 36, 38, 39, 40, 43, 45,* and *46*) whereas 5 of the *CaLTPs* (*CaLTP 7, 12, 30, 44,* and *48*) were downregulated during *H. armigera*-infestation and the rest of them did not alter their expression significantly. *CaLTP45* showed approximately 5-fold upregulation followed by *CaLTP 8* and *36.* The upregulated expression pattern for the remaining *CaLTPs* ranged from 1.5 to 2-fold in response to *H. armigera*-infestation.

### 3.9 Tissue-specific gene expression profile of *CaLTPs*

**FIGURE 8:**
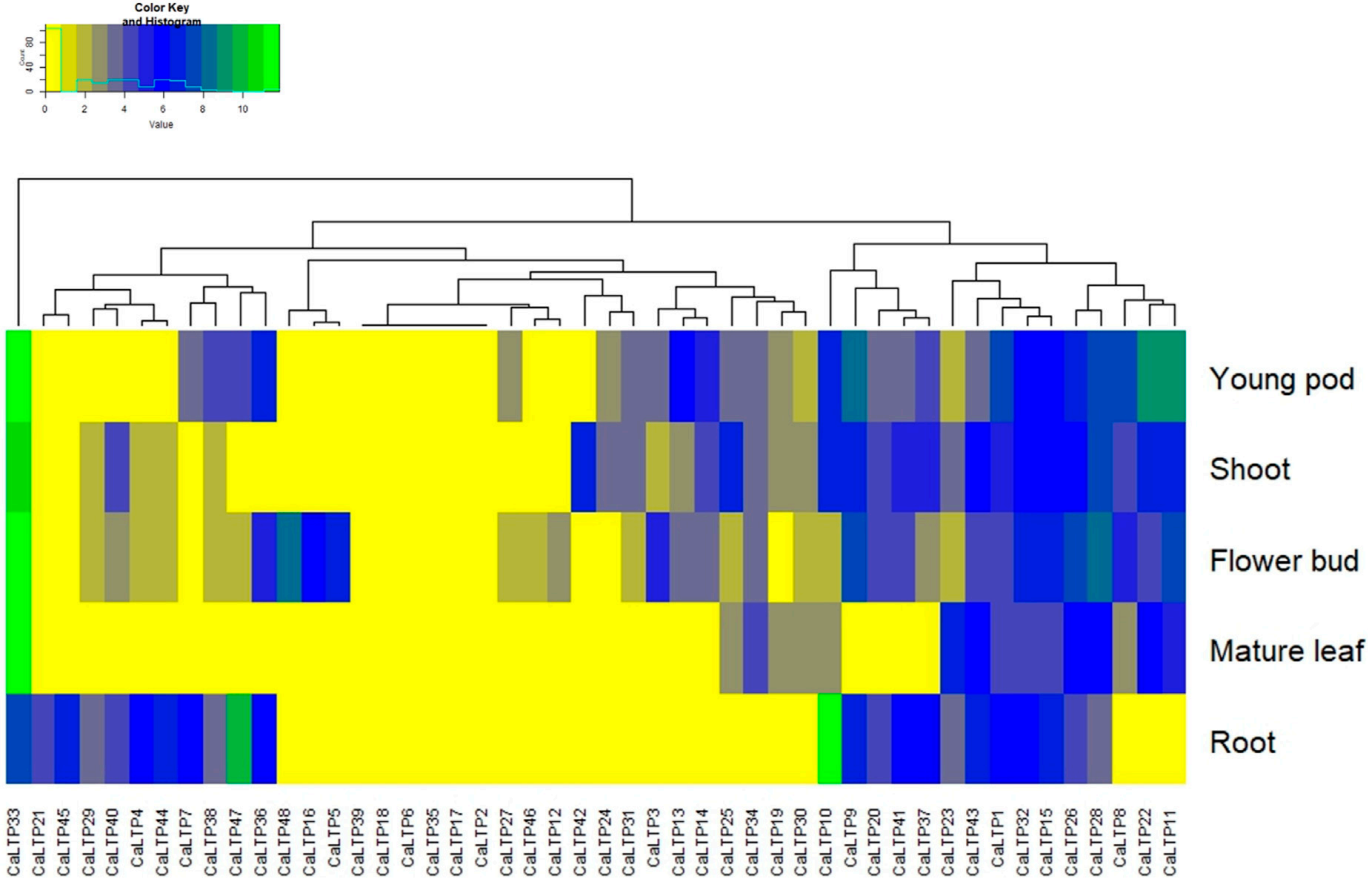
Representation of the gene expression levels of 48 CaLTPs in vegetative and reproductive tissues (shoot, root, mature leaf, flower bud, and young pod) in the form of a heat map.

Expression analysis was performed to study the differential expression of *CaLTPs* in the root, shoot, flower bud, mature leaf, and young pod (Figure 8). The differential expression values were estimated as Reads/Kb/Million (RPKM) values and the heatmap was created with log2 normalized data. It was observed that *CaLTP33* was highly expressed in all the tissue types except the roots. *CaLTP10* was found to be highly expressed whereas *CaLTP47* was expressed mildly in the roots. *CaLTP 9, 11, 22,* and *33* had higher expression in the young pods. *CaLTP 28, 33*, and *48* were found to be expressed in the flower buds. Contrastingly, most of the *CaLTP* genes either showed no expression or exhibited very low levels of expression in the vegetative and reproductive tissues.

## 4 Discussion

*C. arietinum* L.*,* commonly known as chickpea, is widely grown and the second-largest consumed legume in the world. Asia accounts for 89.7% area under cultivation followed by Africa (4.3% area) and occurs as the largest producer in the world (Varshney et al., 2013). However, chickpea faces tremendous pre- and post-harvest damages due to several biotic and abiotic stresses. Among biotic stresses, the damage caused due to insect attacks accounts for 18%–50% of the yield loss (Pandey et al., 2017). Amongst several insects that affect chickpea’s growth and productivity, the pod borer, *H. armigera* is a voracious pest causing a notable decline in the yield and leading to severe economic losses (Romeis et al., 2004). To prevent this, using pesticides is an effective approach but it harms the environment. With the advancement of techniques used to enhance agricultural productivity, researchers have started looking for solutions at the molecular and genetic levels. Thus, the mechanisms underlying the plant-insect interactions are intriguing as it includes identifying the genes that regulate specific responses against herbivory, their expression, and their mode of action. One such gene family which has been known to have functions in plant defense against pests is the non-specific Lipid Transfer Proteins (LTPs); one of the members of the LTP family is reported to be upregulated during *H. armigera* -infestation. Even though it has been a hub for new research in many plants such as *Arabidopsis*, rice, wheat and so on over the past few decades, very little is known about its role in chickpea.

This study encompasses genome-wide identification and characterization of LTPs which were upregulated in *C. arietinum* upon *H. armigera* -infestation. Forty-eight LTPs were identified using *in silico* analysis, and have been structurally and functionally characterized during this study. The assessment of their physiochemical properties revealed the unstable and hydrophilic nature of the LTPs. The subcellular localization of the LTPs suggested that they were localized in extracellular spaces, vacuole, and chloroplast while few were localized in the cytosol, nucleus, and plasma membrane. This coincides with the studies, which show that LTPs are localized in exterior spaces to the plasma membrane (Chou et al., 2006). Furthermore, we attempted to find the locations of these 48 LTPs in the chickpea genome and discovered that the genes were dispersed unevenly on the eight chickpea chromosomes. Most of the genes were present at the distal ends of the chromosomes, which is suggestive of causing divergence of LTPs with each other and with respect to other plant species as well (Bulger and Groudine, 2011).

The promotor analysis of the *LTP* family disclosed promoter elements or motifs specific to *LTP*s have multifarious roles in hormonal regulation, defense and stress mechanisms, and abiotic stress responses. Interestingly, the cis-regulatory elements for light responsiveness were common to all the *LTPs*, and about 65% of the *LTP*s were found to have MeJA-responsive cis-regulatory elements. The presence of MeJA motifs gave the impression of *LTP*s contributing to plant innate immunity and phytohormone pathways. There have been previous reports that endorse the fact that *LTP*s are regulated during plant stress, hormonal signaling, and plant development, and thus, similar roles of *CaLTP*s stipulate the presence of conserved domains for plant defense mechanisms (Wei and Zhong, 2014; Finkina et al., 2016; Wang et al., 2021). The arrangement of introns and exons of *CaLTP*s gave insights into the structural attributes of the 48 genes, such that the introns ranged from 0 to 5 in number, with a greater stretch of exons or CDS sequences in the genes. Since introns in eukaryotic genes contributes to evolutionary advantage, fewer introns in *LTP*s suggested low evolutionary conservation (Gorlova et al., 2014). Moreover, genes with fewer introns are likely to be induced faster in response to external stimuli (Heidari et al., 2022). The identification of 10 novel motifs provided information on the motifs that were common to a majority of the chickpea *LTP*s and the motifs that were linked to each other. Functional annotation of these motifs divulged their involvement in stress responses and lipid transport activities. Thus, the conserved motifs and gene structure of the CaLTPs shared a noteworthy similarity which is suggestive of higher conservation. In addition, CaLTPs were annotated using GO terms under molecular functions, biological processes, and cellular component, categories, suggesting the putative roles of the LTPs in lipid binding and plant defense majorly and localization in the extracellular spaces. Initially LTPs were only associated with inter-membrane lipid transport, yet further studies confirmed their role in defense against biotic and abiotic stress.

Understanding the evolutionary pattern is crucial for the reasons behind the diverse roles exhibited by a gene or a gene family. Phylogenetic analyses of the 48 LTPs with the LTP families of model organisms, namely, *A. thaliana, M. truncatula,* and *O. sativa*, manifested a close relationship of CaLTPs with LTPs from *A. thaliana* and *M. truncatula.* This indicates that LTPs from chickpea are orthologous to those from *A. thaliana* and *M. truncatula*, which may have diverged during speciation events during the evolutionary timeline. Interestingly, the LTP family of rice (a monocot) showed peculiarity in the phylogenetic tree as they were present as distinct clusters with no significant relatedness with any of the CaLTPs and rare instances of relatedness with LTPs from *A. thaliana* and *M. truncatula*, providing strong evidence of evolutionary divergence.

The gene duplication analysis of the *CaLTPs* suggested that both tandem and segmental duplication might have played an important role in the evolution and expansion of the *LTP* family. Further, decrease in the number of orthologous genes between *C. arietinum-M. truncatula* (∼70%), -*A. thaliana* (∼37%), and -*O. sativa* (∼2%) revealed the close relationship of chickpea with other dicots, while having least relatedness with genes from *O. sativa*. This analysis also supports the results of phylogenetic analysis that *CaLTPs* evolved independently of *LTP*s from *O. sativa*, forming a separate clade. The ratio of synonymous and non-synonymous mutations in duplicated gene pairs, or Ka/Ks, revealed the selection pressure on a gene or protein. All of the duplicated gene pairs had a Ka/Ks ratio value of less than 1, indicating that the *CaLTP* genes had undergone purifying selection during the course of evolution, eliminating any harmful alleles (Kondrashov et al., 2002).

The gene expression patterns delineated by RNA-Seq of *CaLTP*s in different tissues such as root, shoot, leaf, pod, and flower buds, yielded profiles showing variation in the expression patterns in vegetative and reproductive tissues, indicating their role in overall growth and development of respective tissue type. However, many of the *CaLTPs* exhibited very low/no expression in different tissue, indicating their role in other processes. The changes at the transcript level during insect herbivory on plants have been extensively studied in several plant systems, such as *A. thaliana, Solanum lycopersicum, Nicotiana attenuata, Gossypium* sp., and so on, which elucidate the complex changes in the expression patterns in signaling and hormonal pathways in response to herbivory. Additionally, gene families involved in MAPK pathway (MAPK, MAPKK, MAPKKK) of chickpea were also investigated, showing the involvement of MAPK pathway in signaling during plant defense against *H. armigera* (Singh et al., 2017; Keshan et al., 2021). In this study, the relative expression patterns of MeJA-regulated *LTP*s were evaluated, which showed their responsiveness towards MeJA and herbivory (Pandey et al., 2017). Interestingly, a positive fold change was observed for 9 of the *CaLTP*s*,* whereas 5 of the *CaLTP*s were downregulated during *H. armigera*-infestation, suggesting differential expression of *CaLTP*s during the *H. armigera -*infestation. Our results supports the notion that *LTP*s are involved in lipid signalling and plant defense against *H. armigera*-infestation.

## 5 Conclusion

The *H. armigera*-infestation on chickpea leads to a surfeit of changes at molecular and biochemical levels. Alteration in the transcript level of defense-related genes such as lipid transfer proteins (LTPs) is one such consequence observed during the attack and is the basis of this genome-wide analysis. We speculate a significant contribution of the *LTP* family of chickpea in plant defense responses and its primary role in lipid transfer activity. Identifying and characterizing 48 *CaLTP*s helped to dissect the genetic expansion, evolution, distribution, and gene expression patterns of these proteins in chickpea. Motif analysis and GO-based annotation highlighted the functional importance of LTPs in various stress responses and plant developmental processes. Differential expression of 14 *CaLTP*s during *H. armigera-*infestation indicated their role in plant defense. This evaluation will not only serve as a fundamental point for further elucidation and validation of functions of LTPs in chickpea and legumes *per se* but will also provide a platform for studying the stress-related pathways and mechanisms with special emphasis on herbivory.

## Data Availability

The original contributions presented in the study are included in the article/Supplementary Material, further inquiries can be directed to the corresponding authors.

## Abbreviations

C, degree Celsius; µg, microgram; ABA, Abscisic Acid; bp, base pairs; CDD, Conserved Domains Database; cDNA, complementary Deoxyribonucleic Acid; CDS, Coding Sequence; C_t_, Cycle threshold; CTDB, Chickpea Transcriptome Database; Da, Dalton; GO, Gene Ontology; GRAVY, GRand Average of hYdropathicity; GSDS, Gene Structure Display Server; iTOL, interactive Tree Of Life, LTP, Lipid Transfer Proteins; MEGA, Molecular Evolutionary Genetic Analysis; MeJA, Methyl Jasmonate; ML, Maximum Likelihood; MSA, Multiple Sequence Alignment; NCBI, National Center for Biotechnology Information; pI, Isoelectric point; qRT-PCR, quantitative Real Time-Polymerase Chain Reaction; RNA, Ribonucleic Acid; RQ, Relative Quantification; SA, Salicylic Acid; TAIR, The Arabidopsis Information Resource; TIGR, The Institute for Genomic Research; Tm, melting Temperature.
