# Supplementary material for: A comprehensive investigation of lipid-transfer proteins from Cicer arietinum disentangles their role in plant defense against Helicoverpa armigera-infestation
Source: Front Genet. 2023 Jun 30;14:1195554. doi: 10.3389/fgene.2023.1195554 (PMC10348895; doi:10.3389/fgene.2023.1195554)
Supplement: Supplementary file 1 [file Table1.DOCX]

**SUPPLEMENTARY MATERIALS**

**A comprehensive investigation of lipid-transfer proteins from *Cicer arietinum* disentangles their role in plant defense against herbivory**

**Harshita Saxena^1,2^, Harshita Negi^1,3^, Radhika Keshan^1^, Amrita Chakraborty^4^, Amit Roy^5*^ Indrakant K. Singh^6*^ and Archana Singh^1, 7*^**

^1^ Department of Botany, Hansraj College, University of Delhi, Delhi, India

^2^ Institute of Plant Breeding, Genetics and Genomics, University of Georgia, Griffin, GA, USA

^3^ Department of Biological Sciences, University of South Carolina, Columbia, SC, USA

^4^  EVA 4.0 Unit, Faculty of Forestry and Wood Sciences, Czech University of Life Sciences Prague, Kamýcká 129, 165 21 Praha 6 – Suchdol.

^5^Forest Molecular Entomology Lab, EXTEMIT-K, EVA 4.0, Faculty of Forestry and Wood Sciences, Czech University of Life Sciences Prague, Kamýcká 129, 165 21 Praha 6 – Suchdol.

^6^Department of Zoology, Deshbandhu College, University of Delhi, New Delhi, India

^7^Delhi School of Climate Change and Sustainability, Institution of Eminence, Maharishi Karnad Bhawan, University of Delhi, Delhi, India.

**
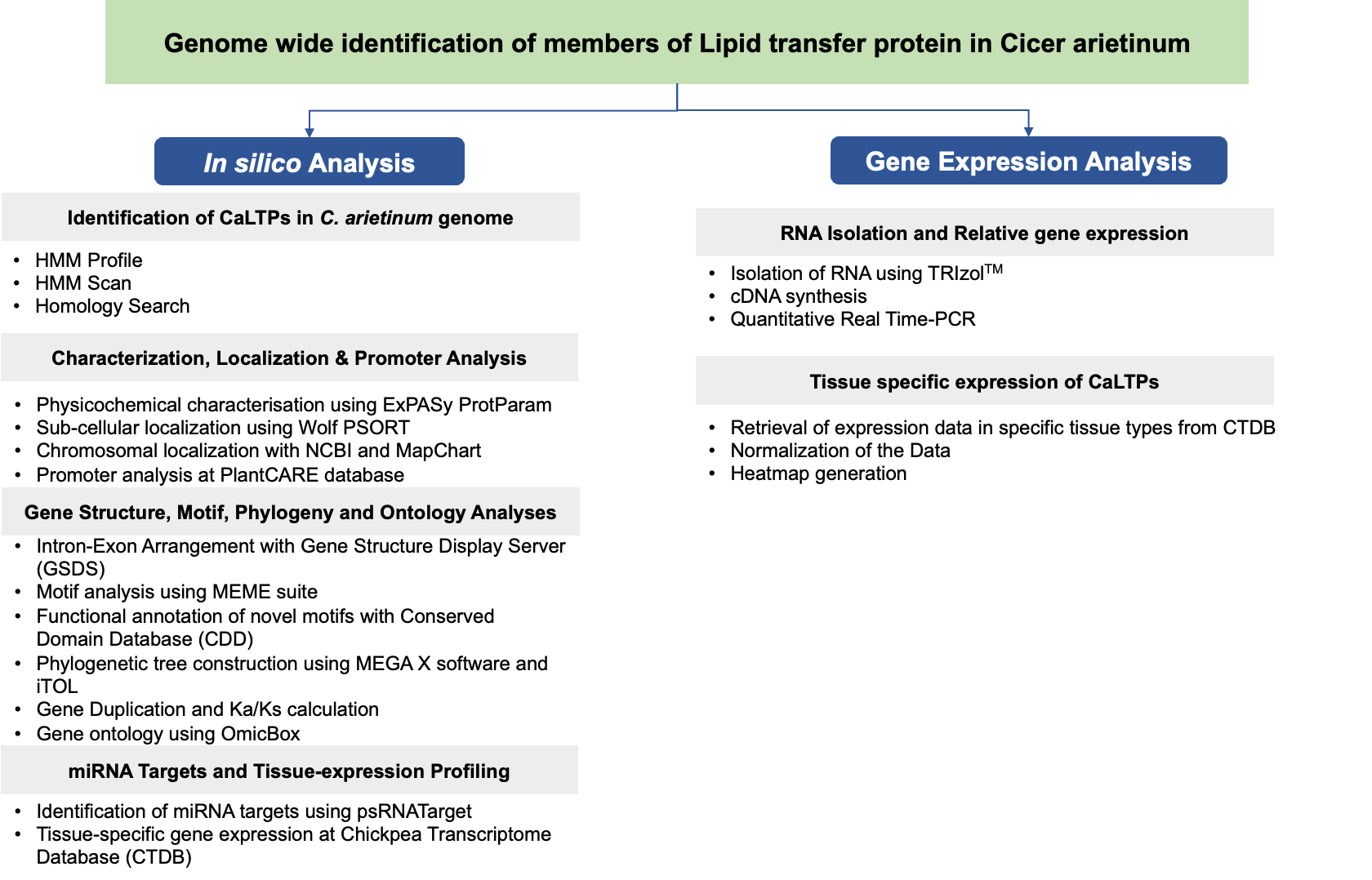
**

**Supplementary Figure 1: Flowchart showing methodology.**

**
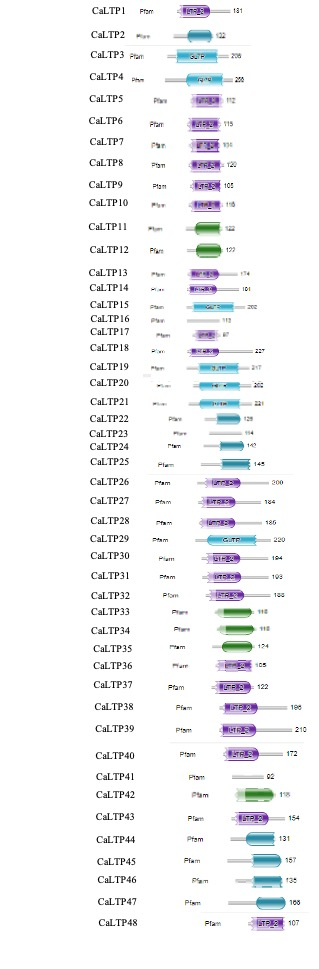
**

**Supplementary Figure 2:** HMMScan result showing presence of conserved LTP domain in CaLTPs

**Supplementary Table 1A:** Nomenclature of LTPs on the basis of chromosomal location

| S.No. | LTP ID | Chr.  No. | Location | Nomenclature |
| --- | --- | --- | --- | --- |
|  | XP_004485560 | Ca1 | NC_021160.1 (984026..985521, complement) | *CaLTP1* |
|  | NP_001266108 | Ca1 | NC_021160.1 (6453091..6453757) | *CaLTP2* |
|  | XP_004486446 | Ca1 | NC_021160.1 (7224384..7228757) | *CaLTP3* |
|  | XP_004486475 | Ca1 | NC_021160.1 (7560316..7562311) | *CaLTP4* |
|  | XP_004487267 | Ca1 | NC_021160.1 (13057788..13058439, complement) | *CaLTP5* |
|  | XP_004488587 | Ca1 | NC_021160.1 (39143859..39144981) | *CaLTP6* |
|  | XP_004491449 | Ca2 | NC_021161.1 (35154335..35155198) | *CaLTP7* |
|  | XP_004492086 | Ca3 | NC_021162.1 (11292316..11293833, complement) | *CaLTP8* |
|  | XP_004492249 | Ca3 | NC_021162.1 (16350967..16351698, complement) | *CaLTP9* |
|  | XP_004492374 | Ca3 | NC_021162.1 (18330024..18331763, complement) | *CaLTP10* |
|  | XP_004492552 | Ca3 | NC_021162.1 (21198302..21199506) | *CaLTP11* |
|  | XP_004492553 | Ca3 | NC_021162.1 (21209739..21211829) | *CaLTP12* |
|  | XP_004493692 | Ca3 | NC_021162.1 (30756156..30757115, complement) | *CaLTP13* |
|  | XP_004495185 | Ca4 | NC_021163.1 (1944437..1945530) | *CaLTP14* |
|  | XP_004495360 | Ca4 | NC_021163.1 (3149170..3153287, complement) | *CaLTP15* |
|  | XP_004496838 | Ca4 | NC_021163.1 (12729622..12730838) | *CaLTP16* |
|  | XP_004496598 | Ca4 | NC_021163.1 (12744435..12745201, complement) | *CaLTP17* |
|  | XP_004496706 | Ca4 | NC_021163.1 (13565423..13567162, complement) | *CaLTP18* |
|  | XP_004497102 | Ca4 | NC_021163.1 (16451158..16454369) | *CaLTP19* |
|  | XP_004497979 | Ca4 | NC_021163.1 (31723122..31733192, complement) | *CaLTP20* |
|  | XP_004498387 | Ca4 | NC_021163.1 (38867369..38871466, complement) | *CaLTP21* |
|  | XP_004498751 | Ca4 | NC_021163.1 (43770058..43771005, complement) | *CaLTP22* |
|  | XP_004500363 | Ca5 | NC_021164.1 (27114135..27115124) | *CaLTP23* |
|  | XP_004501348 | Ca5 | NC_021164.1 (34915339..34915946, complement) | *CaLTP24* |
|  | XP_004501349 | Ca5 | NC_021164.1 (34919517..34920495, complement) | *CaLTP25* |
|  | XP_004501987 | Ca5 | NC_021164.1 (40199046..40203925, complement) | *CaLTP26* |
|  | XP_004502215 | Ca5 | NC_021164.1 (41968037..41970273, complement) | *CaLTP27* |
|  | NP_001265899 | Ca5 | NC_021164.1 (42592043..42595419, complement) | *CaLTP28* |
|  | XP_004502592 | Ca5 | NC_021164.1 (44919901..44921802, complement) | *CaLTP29* |
|  | XP_004504341 | Ca6 | NC_021165.1 (10273722..10274948, complement) | *CaLTP30* |
|  | XP_004504343 | Ca6 | NC_021165.1 (10284583..10285852, complement) | *CaLTP31* |
|  | XP_004504776 | Ca6 | NC_021165.1 (14666486..14668106, complement) | *CaLTP32* |
|  | NP_001296611 | Ca6 | NC_021165.1 (36901105..36901951, complement) | *CaLTP33* |
|  | XP_004506486 | Ca6 | NC_021165.1 (39640427..39643284) | *CaLTP34* |
|  | XP_004506487 | Ca6 | NC_021165.1 (39767649..39769170) | *CaLTP35* |
|  | XP_004507410 | Ca6 | NC_021165.1 (57991604..57992267, complement) | *CaLTP36* |
|  | XP_004509596 | Ca7 | NC_021166.1 (17304163..17305454) | *CaLTP37* |
|  | XP_004511135 | Ca7 | NC_021166.1 (44369099..44371395) | *CaLTP38* |
|  | XP_004511392 | Ca8 | NC_021167.1 (776721..778196, complement) | *CaLTP39* |
|  | XP_004511391 | Ca8 | NC_021167.1 (779268..780562) | *CaLTP40* |
|  | XP_004512892 | Ca8 | NC_021167.1 (14320286..14321078) | *CaLTP41* |
|  | XP_004516173 | Unplaced Scaffold | NW_004516822.1 (82622..83833) | *CaLTP42* |
|  | XP_004515744 | Unplaced Scaffold | NW_004516574.1 (104192..106874) | *CaLTP43* |
|  | XP_004516834 | Unplaced Scaffold | NW_004517997.1 (106980..107718) | *CaLTP44* |
|  | XP_004516836 | Unplaced Scaffold | NW_004517997.1 (116550..117467, complement) | *CaLTP45* |
|  | XP_004516837 | Unplaced Scaffold | NW_004517997.1 (131199..131850) | *CaLTP46* |
|  | XP_004516838 | Unplaced Scaffold | NW_004517997.1 (133583..134436, complement) | *CaLTP47* |
|  | XP_004514935 | Unplaced Scaffold | NW_004516211.1 (202993..204291) | *CaLTP48* |

**Supplementary Table 1B:** Gene duplication in *LTPs* and ka/ks value calculation

| **Duplicated gene pair** | | **ks** | **ka** | **ka/ks** |
| --- | --- | --- | --- | --- |
| **Gene name 1** | **Gene name 2** |  |  |  |
| CaLTP13 | XP_013449762.1 | 0.3183 | 0.1639 | 0.5151 |
| CaLTP9 | XP_003622804.1 | 0.5679 | 0.1841 | 0.3242 |
| CaLTP10 | XP_039684292.1 | 0.5443 | 0.1133 | 0.2081 |
| CaLTP19 | XP_013470196.1 | 0.4277 | 0.103 | 0.2409 |
| CaLTP15 | XP_003590834.1 | 0.1976 | 0.024 | 0.1216 |
| CaLTP17 | XP_003592302.1 | 0.4662 | 0.1345 | 0.2886 |
| CaLTP18 | XP_013469813.1 | 0.4568 | 0.1141 | 0.2498 |
| CaLTP22 | XP_003588779.1 | 0.4054 | 0.132 | 0.3256 |
| CaLTP14 | XP_024635946.1 | 0.4678 | 0.1227 | 0.2622 |
| CaLTP18 | XP_003590611.1 | 14.8586 | 0.6913 | 0.0465 |
| CaLTP14 | XP_013469817.1 | 34.6601 | 0.4337 | 0.0125 |
| CaLTP21 | XP_013466398.1 | 0.2283 | 0.0635 | 0.278 |
| CaLTP20 | XP_013467044.1 | 0.2197 | 0.0432 | 0.1967 |
| CaLTP21 | XP_003602163.1 | 0.6374 | 0.2046 | 0.321 |
| CaLTP22 | XP_003603362.1 | 1.3412 | 0.1913 | 0.1426 |
| CaLTP1 | XP_003593172.3 | 0.3935 | 0.3197 | 0.8126 |
| CaLTP3 | XP_013463001.1 | 0.3365 | 0.1002 | 0.2978 |
| CaLTP4 | XP_003594554.1 | 0.2256 | 0.1021 | 0.4524 |
| CaLTP2 | XP_003594279.1 | 0.3534 | 0.1713 | 0.4848 |
| CaLTP24 | XP_003588784.3 | 1.0888 | 0.2781 | 0.2554 |
| CaLTP29 | XP_013466398.1 | 0.6091 | 0.1921 | 0.3154 |
| CaLTP26 | XP_003601303.1 | 0.8288 | 0.3486 | 0.4206 |
| CaLTP27 | XP_024633668.1 | 0.3641 | 0.1325 | 0.3639 |
| CaLTP28 | XP_003601770.1 | 0.6767 | 0.2022 | 0.2988 |
| CaLTP29 | XP_003602163.1 | 0.1719 | 0.0543 | 0.316 |
| CaLTP23 | XP_013460446.1 | 0.324 | 0.08 | 0.247 |
| CaLTP24 | XP_003603362.1 | 0.4321 | 0.1481 | 0.3428 |
| CaLTP27 | XP_003629697.1 | 1.1502 | 0.3618 | 0.3146 |
| CaLTP32 | XP_003593172.3 | 1.8419 | 0.3331 | 0.1809 |
| CaLTP36 | XP_013456374.1 | 0.3955 | 0.2306 | 0.583 |
| CaLTP33 | XP_039688589.1 | 0.7618 | 0.6224 | 0.817 |
| CaLTP35 | XP_003605293.2 | 0.2931 | 0.1081 | 0.3687 |
| CaLTP30 | XP_003629697.1 | 0.4122 | 0.1529 | 0.3709 |
| CaLTP37 | XP_024628822.2 | 0.2863 | 0.1029 | 0.3593 |
| CaLTP38 | XP_013445286.1 | 0.4222 | 0.1065 | 0.2522 |
| CaLTP39 | XP_013469813.1 | 0.8536 | 0.3136 | 0.3673 |
| CaLTP40 | XP_013469817.1 | 0.9335 | 0.2324 | 0.2489 |
| CaLTP39 | XP_003610780.1 | 0.2987 | 0.1778 | 0.5952 |
| CaLTP40 | XP_003610781.1 | 0.2148 | 0.1112 | 0.5177 |
| CaLTP41 | XP_003620406.1 | 0.4286 | 0.0526 | 0.1227 |
| CaLTP43 | XP_013457632.1 | 0.8583 | 0.2455 | 0.286 |
| CaLTP45 | XP_003608743.1 | 0.3762 | 0.0973 | 0.2586 |
| CaLTP46 | XP_003608746.1 | 0.4573 | 0.0543 | 0.1187 |

**Supplementary Table 2:** The role of different promoter elements of CaLTPs in hormonal, defense and stress responses, as well as plant developmental processes.

| Name of gene | Auxin | Gibberellin | ABA | SA | MeJA | Elicitor-mediated activation | Defense and stress responsiveness | Light responsiveness | Drought-inducibility | Low-temperature response | Anaerobic induction | Meristem expression | Endosperm expression | Zein metabolism expression | Circadian control | Cell Cycle |
| --- | --- | --- | --- | --- | --- | --- | --- | --- | --- | --- | --- | --- | --- | --- | --- | --- |
| *CaLTP1* | 0 | 0 | 0 | 0 | 0 | 0 | 0 | 4 | 0 | 0 | 0 | 1 | 0 | 0 | 0 | 0 |
| *CaLTP2* | 0 | 1 | 0 | 1 | 0 | 0 | 0 | 5 | 0 | 1 | 0 | 0 | 0 | 0 | 0 | 0 |
| *CaLTP3* | 0 | 0 | 1 | 1 | 0 | 0 | 0 | 5 | 1 | 0 | 0 | 0 | 0 | 1 | 0 | 0 |
| *CaLTP4* | 1 | 0 | 0 | 0 | 0 | 0 | 0 | 8 | 0 | 0 | 0 | 0 | 0 | 0 | 0 | 0 |
| *CaLTP5* | 0 | 0 | 1 | 1 | 2 | 0 | 0 | 6 | 0 | 0 | 1 | 0 | 0 | 1 | 0 | 0 |
| *CaLTP6* | 1 | 0 | 0 | 0 | 0 | 0 | 0 | 6 | 0 | 0 | 0 | 0 | 0 | 0 | 0 | 0 |
| *CaLTP7* | 0 | 0 | 0 | 0 | 2 | 0 | 0 | 5 | 1 | 0 | 1 | 1 | 0 | 0 | 1 | 0 |
| *CaLTP8* | 0 | 0 | 0 | 0 | 2 | 0 | 1 | 3 | 0 | 0 | 0 | 2 | 0 | 0 | 0 | 0 |
| *CaLTP9* | 0 | 0 | 1 | 1 | 2 | 0 | 0 | 6 | 0 | 0 | 0 | 0 | 0 | 1 | 0 | 0 |
| *CaLTP10* | 0 | 0 | 0 | 0 | 0 | 0 | 0 | 6 | 1 | 0 | 0 | 0 | 0 | 1 | 0 | 0 |
| *CaLTP11* | 0 | 0 | 0 | 0 | 0 | 0 | 0 | 7 | 0 | 0 | 1 | 0 | 0 | 0 | 0 | 0 |
| *CaLTP12* | 2 | 1 | 2 | 0 | 4 | 0 | 0 | 10 | 0 | 0 | 1 | 0 | 0 | 0 | 0 | 0 |
| *CaLTP13* | 0 | 0 | 0 | 0 | 0 | 0 | 0 | 11 | 0 | 0 | 2 | 0 | 1 | 1 | 0 | 0 |
| *CaLTP14* | 1 | 1 | 0 | 0 | 0 | 0 | 0 | 4 | 1 | 0 | 1 | 0 | 1 | 0 | 0 | 0 |
| *CaLTP15* | 1 | 1 | 0 | 0 | 0 | 1 | 0 | 17 | 0 | 0 | 0 | 0 | 0 | 0 | 0 | 0 |
| *CaLTP16* | 0 | 0 | 1 | 0 | 0 | 0 | 1 | 4 | 0 | 0 | 1 | 1 | 0 | 0 | 1 | 0 |
| *CaLTP17* | 0 | 0 | 1 | 0 | 0 | 0 | 0 | 5 | 0 | 0 | 0 | 1 | 1 | 0 | 0 | 0 |
| *CaLTP18* | 1 | 0 | 1 | 0 | 4 | 0 | 1 | 5 | 0 | 0 | 0 | 1 | 0 | 0 | 1 | 0 |
| *CaLTP19* | 0 | 0 | 0 | 0 | 0 | 0 | 0 | 6 | 0 | 0 | 0 | 0 | 0 | 0 | 1 | 1 |
| *CaLTP20* | 0 | 0 | 0 | 1 | 0 | 0 | 0 | 3 | 0 | 0 | 1 | 0 | 0 | 0 | 1 | 0 |
| *CaLTP21* | 0 | 0 | 0 | 0 | 0 | 0 | 1 | 6 | 0 | 0 | 2 | 0 | 0 | 0 | 0 | 0 |
| *CaLTP22* | 0 | 0 | 2 | 0 | 2 | 0 | 0 | 11 | 0 | 0 | 4 | 0 | 0 | 0 | 0 | 0 |
| *CaLTP23* | 1 | 0 | 0 | 1 | 0 | 1 | 1 | 3 | 0 | 0 | 1 | 1 | 1 | 0 | 0 | 0 |
| *CaLTP24* | 0 | 0 | 0 | 1 | 2 | 0 | 0 | 4 | 0 | 0 | 1 | 0 | 0 | 0 | 0 | 0 |
| *CaLTP25* | 0 | 0 | 0 | 0 | 0 | 0 | 1 | 1 | 0 | 0 | 0 | 0 | 0 | 0 | 0 | 0 |
| *CaLTP26* | 0 | 1 | 0 | 1 | 0 | 0 | 1 | 6 | 0 | 0 | 1 | 0 | 0 | 1 | 0 | 0 |
| *CaLTP27* | 0 | 0 | 1 | 0 | 0 | 0 | 2 | 9 | 0 | 0 | 0 | 0 | 0 | 0 | 0 | 0 |
| *CaLTP28* | 0 | 0 | 0 | 0 | 2 | 0 | 0 | 6 | 0 | 0 | 2 | 0 | 0 | 0 | 0 | 0 |
| *CaLTP29* | 0 | 3 | 1 | 0 | 0 | 0 | 0 | 9 | 0 | 0 | 0 | 0 | 0 | 0 | 0 | 0 |
| *CaLTP30* | 0 | 0 | 1 | 0 | 2 | 0 | 0 | 7 | 1 | 0 | 0 | 1 | 0 | 1 | 1 | 0 |
| *CaLTP31* | 0 | 0 | 1 | 0 | 2 | 0 | 0 | 7 | 0 | 0 | 1 | 0 | 0 | 1 | 0 | 0 |
| *CaLTP32* | 1 | 0 | 0 | 0 | 0 | 0 | 0 | 6 | 0 | 0 | 1 | 0 | 0 | 0 | 0 | 0 |
| *CaLTP33* | 0 | 1 | 0 | 0 | 2 | 0 | 0 | 6 | 0 | 1 | 0 | 0 | 0 | 0 | 0 | 0 |
| *CaLTP34* | 1 | 0 | 0 | 0 | 0 | 0 | 0 | 1 | 0 | 0 | 0 | 0 | 0 | 0 | 0 | 0 |
| *CaLTP35* | 0 | 0 | 2 | 0 | 0 | 0 | 0 | 9 | 0 | 0 | 1 | 0 | 0 | 0 | 0 | 0 |
| *CaLTP36* | 0 | 0 | 1 | 0 | 2 | 0 | 1 | 9 | 0 | 0 | 3 | 0 | 0 | 0 | 0 | 0 |
| *CaLTP37* | 1 | 1 | 2 | 0 | 0 | 0 | 0 | 2 | 0 | 0 | 2 | 0 | 0 | 0 | 0 | 0 |
| *CaLTP38* | 0 | 1 | 1 | 1 | 2 | 0 | 1 | 4 | 0 | 0 | 1 | 0 | 0 | 2 | 0 | 0 |
| *CaLTP39* | 1 | 0 | 0 | 0 | 2 | 0 | 0 | 4 | 0 | 0 | 1 | 0 | 1 | 0 | 0 | 0 |
| *CaLTP40* | 1 | 0 | 2 | 0 | 2 | 0 | 1 | 5 | 0 | 0 | 1 | 0 | 0 | 0 | 1 | 0 |
| *CaLTP41* | 0 | 1 | 0 | 1 | 0 | 0 | 1 | 6 | 1 | 2 | 0 | 1 | 1 | 1 | 0 | 0 |
| *CaLTP42* | 1 | 1 | 1 | 1 | 0 | 1 | 0 | 4 | 0 | 0 | 0 | 0 | 0 | 0 | 1 | 0 |
| *CaLTP43* | 0 | 1 | 0 | 0 | 2 | 0 | 1 | 4 | 0 | 0 | 1 | 0 | 1 | 0 | 0 | 0 |
| *CaLTP44* | 0 | 0 | 1 | 0 | 2 | 0 | 0 | 8 | 0 | 0 | 2 | 0 | 1 | 0 | 0 | 0 |
| *CaLTP45* | 0 | 0 | 1 | 0 | 2 | 0 | 2 | 7 | 0 | 1 | 1 | 1 | 0 | 0 | 0 | 0 |
| *CaLTP46* | 2 | 0 | 0 | 0 | 2 | 0 | 0 | 11 | 0 | 0 | 3 | 0 | 0 | 0 | 1 | 0 |
| *CaLTP47* | 1 | 0 | 2 | 1 | 0 | 1 | 0 | 6 | 0 | 0 | 0 | 0 | 0 | 0 | 0 | 0 |
| *CaLTP48* | 0 | 1 | 0 | 0 | 2 | 0 | 0 | 6 | 0 | 0 | 3 | 0 | 0 | 0 | 0 | 0 |

**Supplementary Figure 3:** Presence or absence of promoter elements responsible for plant development and external responses in different LTPs.


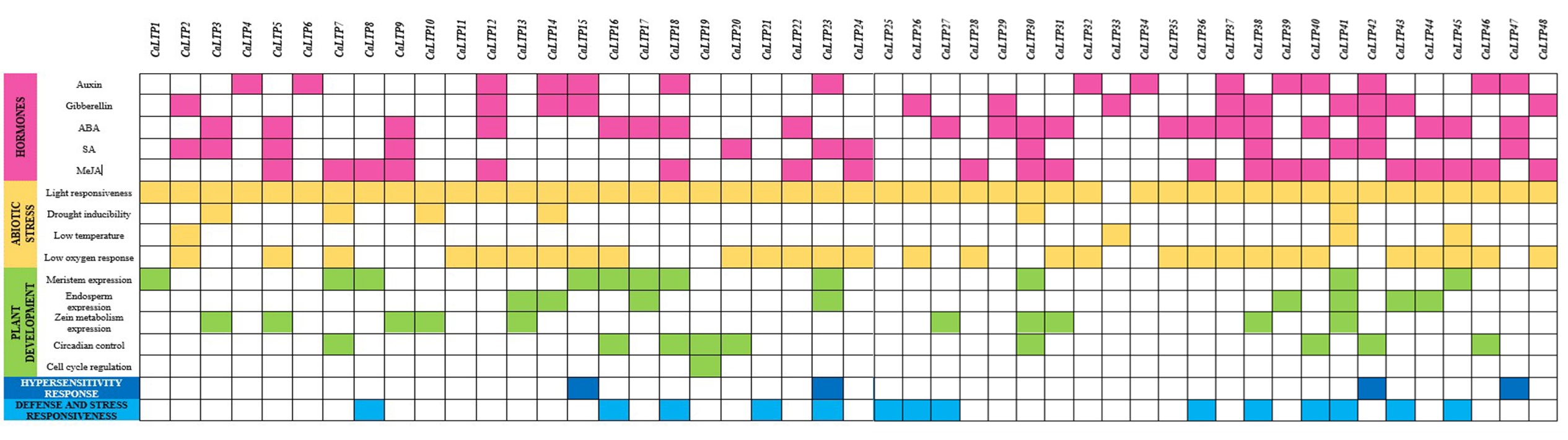


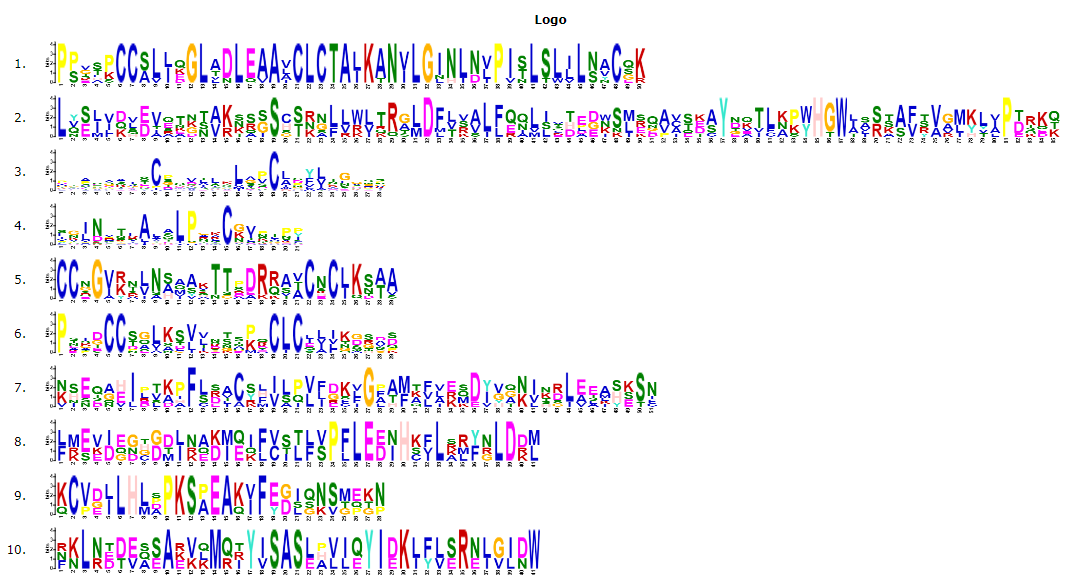


**Supplementary Figure 4:** WebLogo showing the consensus sequences of the 10 novel conserved motifs that were identified in the LTP gene family of chickpea using MEME suite.

**Supplementary Table 3:** The ten novel motifs identified in 48 CaLTPs and the enriched motifs in Arabidopsis thaliana, Medicago truncatula, and Oryza sativa.

| Motifs | | | | | |
| --- | --- | --- | --- | --- | --- |
| Motif Number | **Width** | **Chickpea Motif Sequence** | | **Best Possible Match** | |
| 1 | 50 | PPVSPCCSLJEGLADLEAAVCLCTAJKANVLGINLNVPISLSLILNACQK | | PPVSPCCSLJQGLADLEAAVCLCTAJKANVLGINLNVPISLSLJLNACQK | |
| 2 | 85 | LYSJIDLEVETNTAKSSGSCSRNLLWLTRGLDFLRALFQQLJKTEDKSLRQAVSEAYBKTLKPWHGWIASSAFSVGMKLVPDRKQ | | LVSJVDLEVETGTAKSSSSCSRNLLWLTRGLDFLSALFZQLISTEDKSLSQAASKAYNKTLKPWHGWLSSSAFSVGMKLAPTRKQ | |
| 3 | 28 | PTASAAISCPSVLLKLAPCLSYLTGGSS | | PPASAAISCPSVLLKLAPCLSYLTGGSS | |
| 4 | 21 | LGINVTLALALPAKCGVNTPY | | SGJNVTLALALPAKCGVPTPP | |
| 5 | 29 | CCNGVKNLNSAAKTTPDRRAACNCLKSAA | | CCNGVKNLNSAAKTTPDRRAACNCJKSAA | |
| 6 | 29 | PSIDCCSGLKSVVNSSPQCLCLLIKDSDD | | PSIDCCSGLKSVLNSSPQCLCLLIKGSAS | |
| 7 | 51 | KSEQAEIPTKPFLDACSLILPVFDKLGPAMKFVEQDIVANINRLEEAHKSN | | KSEQAEILTKPFLSACSLILPVFDKLGAAMAFLESDIVANIKRLEEASKSN | |
| 8 | 41 | FMEVDEGGGDJNADIZIFVSLFVPFLEDNHKFLKRYNLDDL | | LMEVIEGTGDJNAKIZIFVSLLSPFLEDIHKFLSRFNLDDL | |
| 9 | 28 | KCVDJLHLPPKSPEAKIFEGIQNSMEKN | | KCVDJLHLSPKSAEAKVFEGIQNSMEKN | |
| 10 | 41 | RKLNDDEASAKVQMQTYISASLPVIQYIDKLFLSRNLGIDW | | NKLNTDESSAKVLMQTYISASLAVJZYIDKJFLSRNJGJBW | |
| Motif Enrichment Analysis | | | | | |
| Enriched chickpea motif in *Arabidopsis thaliana* | | | **Enriched chickpea motif in *Medicago truncatula*** | | **Enriched chickpea motif in *Oryza sativa*** |
| LGINVTLALALPAKCGVNTPY | | | LGINVTLALALPAKCGVNTP | | CCNGVKNLNSAAKTTPDRRAACNCLKSAA |
| PTASAAISCPSVLLKLAPCLSYLTGGSS | | | CCNGVKNLNSAAKTTPDRRAACNCLKSAA | | PTASAAISCPSVLLKLAPCLSYLTGGSS |
| CCNGVKNLNSAAKTTPDRRAACNCLKSAA | | | PTASAAISCPSVLLKLAPCLSYLTGGSS | | LGINVTLALALPAKCGVNTPY |
| PSIDCCSGLKSVVNSSPQCLCLLIKDSDD | | | PSIDCCSGLKSVVNSSPQCLCLLIKDSDD | | PPVSPCCSLJEGLADLEAAVCLCTAJKANVLGINLNVPISLSLILNACQK |
| KCVDJLHLPPKSPEAKIFEGIQNSMEKN | | | PPVSPCCSLJEGLADLEAAVCLCTAJKANVLGINLNVPISLSLILNACQK | |  |

**Supplementary Table 4:** Motif Annotation and GO based functional characterization of 10 LTP motifs using CDD

| Motif  No. | Accession | Superfamily | Description | Gene Ontology | | |
| --- | --- | --- | --- | --- | --- | --- |
|  |  |  |  | **Biological Process** | **Molecular function** | **Cellular Component** |
| 1 | cd01958 | AAI_LTSS | AAI_LTSS: Alpha-Amylase Inhibitors (AAI), Lipid Transfer (LT) and Seed Storage (SS) Protein family; HPS_like: Hydrophobic Protein from Soybean (HPS)-like subfamily | -- | -- | -- |
| 2 | pfam08718 | GLTP | Glycolipid transfer protein (GLTP) ;GLTP is a cytosolic protein that catalyzes the intermembrane transfer of glycolipids. | Cell death; response to salicylic acid; ER to golgi ceramide transport; defense response to bacterium; intermembrane lipid transfer; | Protein binding; glycolipid transfer activity; glycolipid binding; phosphatidylinositol-4-phosphate binding; ceramide binding; sphingomyelin transfer activity; | Nucleoplasm; trans-Golgi network; cytosol; plasma membrane; membrane |
| 3 | -- | -- | -- | -- | -- | -- |
| 4 | -- | -- | -- | -- | -- | -- |
| 5 | cl07890 | AAI_LTSS | AAI_LTSS: Alpha-Amylase Inhibitors (AAI), Lipid Transfer (LT) and Seed Storage (SS) Protein family; | Lipid transport; killing of cells of other organism; defense response to bacterium; defense response to fungus; | Lipid binding; | Membrane |
| 6 | cd00010 | AAI_LTSS | AAI_LTSS: Alpha-Amylase Inhibitors (AAI), Lipid Transfer (LT) and Seed Storage (SS) Protein family | -- | -- | -- |
| 7 | cl07367 | GLTP | Glycolipid transfer protein (GLTP) ;GLTP is a cytosolic protein that catalyzes the intermembrane transfer of glycolipids. | Intermembrane lipid transfer; | Lipid transfer activity; | Cytoplasm; cytosol; plasma membrane |
| 8 | -- | -- | -- | -- | -- | Cytosol; plasma membrane |
| 9 | -- | -- | -- | -- | -- | -- |
| 10 | -- | -- | -- | Response to acid chemical; cell death; response to hormone; response to salicylic acid; organic cation transport; polyol transport; response to drug; defense response to bacterium; innate immune response; response to antibiotic; multi-organism process; ammonium transmembrane transport; intermembrane lipid transfer; ceramide 1-phosphate transport; | Protein binding; ammonium transmembrane transporter activity; organic cation transmembrane transporter activity; polyol transmembrane transporter activity; alcohol transmembrane transporter activity; glycolipid binding; sphingomyelin transfer activity; ceramide 1-phosphate binding; ceramide 1-phosphate transfer activity; | Cytosol; membrane |

**Supplementary Table 5:** Gene-ontology based functional annotation of *CaLTPs* performed using Blast2GO

|  | GO ID | GO: Name | Sequences |
| --- | --- | --- | --- |
| Molecular Functions | GO:0015665 | Alcohol transmembrane transporter activity | CaLTP3 |
|  | GO:0008519 | Ammonium transmembrane transporter activity | CaLTP3 |
|  | GO:0043168 | Anion binding | CaLTP21, CaLTP29 |
|  | GO:0005516 | Calmodulin binding | CaLTP8, CaLTP42, CaLTP34, CaLTP33 |
|  | GO:1902387 | Ceramide 1-phosphate binding | CaLTP4, CaLTP3 |
|  | GO:1902388 | Ceramide 1-phosphate transfer activity | CaLTP4, CaLTP3 |
|  | GO:0097001 | Ceramide binding | CaLTP15, CaLTP21, CaLTP29, CaLTP20 |
|  | GO:0005504 | Fatty acid binding | CaLTP9, CaLTP36, CaLTP7, CaLTP37, CaLTP48, CaLTP10 |
|  | GO:0051861 | Glycolipid binding | CaLTP15, CaLTP21, CaLTP29, CaLTP3 |
|  | GO:0017089 | Glycolipid transfer activity | CaLTP21, CaLTP29, CaLTP15 |
|  | GO:0008289 | Lipid binding | CaLTP8, CaLTP6, CaLTP5, CaLTP1, CaLTP28, CaLTP39, CaLTP14, CaLTP13, CaLTP35, CaLTP16, CaLTP38, CaLTP26, CaLTP32, CaLTP42, CaLTP12, CaLTP23, CaLTP34, CaLTP11, CaLTP33, CaLTP40 |
|  | GO:0120013 | Lipid transfer activity | CaLTP20 |
|  | GO:0005319 | Lipid transporter activity | CaLTP8, CaLTP42, CaLTP34, CaLTP33 |
|  | GO:0045735 | Nutrient reservoir activity | CaLTP8, CaLTP35, CaLTP6, CaLTP42, CaLTP12, CaLTP23, CaLTP34, CaLTP11, CaLTP33 |
|  | GO:0015101 | Organic cation transmembrane transporter activity | CaLTP3 |
|  | GO:0070273 | Phosphatidylinositol-4-phosphate binding | CaLTP21, CaLTP29, CaLTP15 |
|  | GO:0005543 | Phospholipid binding | CaLTP20, CaLTP19 |
|  | GO:0005548 | Phospholipid transporter activity | CaLTP17 |
|  | GO:0015166 | Polyol transmembrane transporter activity | CaLTP3 |
|  | GO:0005515 | Protein binding | CaLTP15, CaLTP4, CaLTP20, CaLTP3, CaLTP19 |
|  | GO:0043621 | Protein self-association | CaLTP9, CaLTP36, CaLTP7 |
|  | GO:0140338 | Sphingomyelin transfer activity | CaLTP4, CaLTP3, CaLTP19 |
|  | GO:0008270 | Zinc ion binding | CaLTP9, CaLTP36, CaLTP7 |
| Biological processes | GO:0072488 | Ammonium transmembrane transport | CaLTP3 |
|  | GO:0008219 | Cell death | CaLTP4, CaLTP3, CaLTP19 |
|  | GO:0032989 | Cellular component morphogenesis | CaLTP8, CaLTP42, CaLTP34, CaLTP33 |
|  | GO:0035627 | Ceramide transport | CaLTP4, CaLTP20, CaLTP3, CaLTP19 |
|  | GO:0042335 | Cuticle development | CaLTP8, CaLTP26, CaLTP42, CaLTP28, CaLTP34, CaLTP33 |
|  | GO:0042742 | Defense response to bacterium | CaLTP4, CaLTP3, CaLTP19 |
|  | GO:0050832 | Defense response to fungus | CaLTP47, CaLTP8, CaLTP35, CaLTP6, CaLTP42, CaLTP12, CaLTP34, CaLTP11, CaLTP33, CaLTP28 |
|  | GO:0050830 | Defense response to Gram-positive bacterium | CaLTP8, CaLTP35, CaLTP6, CaLTP42, CaLTP12, CaLTP34, CaLTP11, CaLTP33 |
|  | GO:0098542 | Defense response to other organism | CaLTP23 |
|  | GO:0035621 | ER to Golgi ceramide transport | CaLTP21, CaLTP29, CaLTP15 |
|  | GO:0046836 | Glycolipid transport | CaLTP21, CaLTP29 |
|  | GO:0045087 | Innate immune response | CaLTP3 |
|  | GO:0120009 | Intermembrane lipid transfer | CaLTP15, CaLTP21, CaLTP4, CaLTP20, CaLTP3, CaLTP29, CaLTP19 |
|  | GO:0010102 | Lateral root morphogenesis | CaLTP47 |
|  | GO:0006869 | Lipid transport | CaLTP8, CaLTP6, CaLTP5, CaLTP1, CaLTP28, CaLTP39, CaLTP14, CaLTP13, CaLTP35, CaLTP16, CaLTP38, CaLTP26, CaLTP32, CaLTP42, CaLTP12, CaLTP23, CaLTP34, CaLTP11, CaLTP33, CaLTP41, CaLTP40 |
|  | GO:0051704 | Multi-organism process | CaLTP47, CaLTP28, CaLTP3 |
|  | GO:0048519 | Negative regulation of biological process | CaLTP4, CaLTP20, CaLTP3 |
|  | GO:0015711 | Organic anion transport | CaLTP8, CaLTP42, CaLTP17, CaLTP34, CaLTP33 |
|  | GO:0015695 | Organic cation transport | CaLTP3 |
|  | GO:0006649 | Phospholipid transfer to membrane | CaLTP8, CaLTP42, CaLTP17, CaLTP34, CaLTP33 |
|  | GO:0015914 | Phospholipid transport | CaLTP4, CaLTP3 |
|  | GO:0090627 | Plant epidermal cell differentiation | CaLTP8, CaLTP42, CaLTP34, CaLTP33 |
|  | GO:0015791 | Polyol transport | CaLTP3 |
|  | GO:1901957 | Regulation of cutin biosynthetic process | CaLTP8, CaLTP42, CaLTP34, CaLTP33 |
|  | GO:0001101 | Response to acid chemical | CaLTP3 |
|  | GO:0046677 | Response to antibiotic | CaLTP3 |
|  | GO:0009733 | Response to auxin | CaLTP47 |
|  | GO:0009725 | Response to hormone | CaLTP3 |
|  | GO:0009751 | Response to salicylic acid | CaLTP4, CaLTP3, CaLTP19 |
|  | GO:0009414 | Response to water deprivation | CaLTP8, CaLTP42, CaLTP34, CaLTP33 |
|  | GO:0048316 | Seed development | CaLTP8, CaLTP35, CaLTP6, CaLTP42, CaLTP12, CaLTP23, CaLTP34, CaLTP11, CaLTP33 |
|  | GO:0090378 | Seed trichome elongation | CaLTP8, CaLTP42, CaLTP34, CaLTP33 |
|  | GO:0009627 | Systemic acquired resistance | CaLTP10, CaLTP37, CaLTP48 |
|  | GO:0009862 | Systemic acquired resistance, salicylic acid mediated signalling pathway | CaLTP9, CaLTP36, CaLTP7 |
|  | GO:0042493 | Response to drug | CaLTP3 |
| Cellular Component | GO:0031225 | Anchored component of membrane | CaLTP38 |
|  | GO:0046658 | Anchored component of plasma membrane | CaLTP14, CaLTP1, CaLTP26, CaLTP40, CaLTP32, CaLTP28 |
|  | GO:0048046 | Apoplast | CaLTP8, CaLTP42, CaLTP34, CaLTP33 |
|  | GO:0098588 | Bounding membrane of organelle | CaLTP20, CaLTP15 |
|  | GO:0009707 | Chloroplast outer membrane | CaLTP47 |
|  | GO:0009534 | Chloroplast thylakoid | CaLTP8, CaLTP42, CaLTP34, CaLTP33 |
|  | GO:0005737 | Cytoplasm | CaLTP19 |
|  | GO:0005829 | Cytosol | CaLTP15, CaLTP4, CaLTP20, CaLTP3 |
|  | GO:0005783 | Endoplasmic reticulum | CaLTP9, CaLTP36, CaLTP47, CaLTP7, CaLTP28 |
|  | GO:0005768 | Endosome | CaLTP8, CaLTP42, CaLTP34, CaLTP33 |
|  | GO:0043245 | Extra-organismal space | CaLTP8, CaLTP35, CaLTP6, CaLTP42, CaLTP12, CaLTP23, CaLTP34, CaLTP11, CaLTP33 |
|  | GO:0005794 | Golgi apparatus | CaLTP20, CaLTP28 |
|  | GO:0016020 | Membrane | CaLTP35, CaLTP16, CaLTP6, CaLTP5, CaLTP4, CaLTP42, CaLTP3, CaLTP33, CaLTP19 |
|  | GO:0005654 | Nucleoplasm | CaLTP21, CaLTP29, CaLTP15 |
|  | GO:0005634 | Nucleus | CaLTP20, CaLTP19 |
|  | GO:0009505 | Plant-type cell wall | CaLTP8, CaLTP42, CaLTP28, CaLTP34, CaLTP33 |
|  | GO:0005886 | Plasma membrane | CaLTP8, CaLTP38, CaLTP15, CaLTP20, CaLTP42, CaLTP34, CaLTP33 |
|  | GO:0009506 | Plasmodesma | CaLTP9, CaLTP36, CaLTP47, CaLTP8, CaLTP7, CaLTP42, CaLTP34, CaLTP33 |
|  | GO:0005802 | Trans-Golgi network | CaLTP8, CaLTP15, CaLTP21, CaLTP42, CaLTP34, CaLTP33, CaLTP29 |

**Supplementary Table 6:** Primer sequences designed using the Primer3 tool for the LTPs that showed responsiveness towards MeJA

| S. No. | Name of the Primer | Sequence of the primer |
| --- | --- | --- |
| 1 | CaLTP5F | GAATCTGACCCCACAACTAGG |
| 2 | CaLTP5R | TGCTATTGGAAATTGGGAAGGA |
| 3 | CaLTP7For | GATGCAGTGGATGGTAGCAG |
| 4 | CaLTP7Rev | TTGTATGTGCAGAGGCAAGG |
| 5 | CaLTP8F | GGTCGTTACTCAGCACCATCA |
| 6 | CaLTP8R | ACACCACACCTCCTAGCAAGT |
| 7 | CaLTP9F | TAAGCCAGCAGTGACTCAGC |
| 8 | CaLTP9R | GCAGGAAGTGAAGAAGCAAGA |
| 9 | CaLTP12F | GCGTTGGTTATCTAAGGAGCCC |
| 10 | CaLTP12R | AGCAGGAAGAGCAGCAAGTG |
| 11 | CaLTP18F | ACCACTGAATGCTGCTCTGC |
| 12 | CaLTP18R | GGTGAACCTGAGGCTTTGCA |
| 13 | CaLTP22F | TGGTCATTGCAAGCATACTCCA |
| 14 | CaLTP22R | GCCAAACCTTGAAGCAATGCA |
| 15 | CaLTP24F | TGTCCTACTCCAGCTTCCGC |
| 16 | CaLTP24R | AGTGCAGCCTCCAAATCAGC |
| 17 | CaLTP28F | TGGCTCAGAAATGTGGACAAGT |
| 18 | CaLTP28R | CCCAAACTCTTACTTTCAGGGCT |
| 19 | CaLTP30F | TGTCACTCTTGCCCTCCAGT |
| 20 | CaLTP30R | TCCAGTGGAATTACTAGCCGGA |
| 21 | CaLTP31F | GCACTGGCGTCAAAGATGTTATT |
| 22 | CaLTP31R | GCTTCAGGAGATTTAGGTGCCA |
| 23 | CaLTP36 | GGGGATAATGTTGTTGGCCA |
| 24 | CaLTP36R | AAGCAACCCTGAATCCATGT |
| 25 | CaLTP38For | CAGTTTGTAATGCCCCTGCT |
| 26 | CaLTP38Rev | CCCTTCTTCTTGCTGAATGC |
| 27 | CaLTP39For | GCCTGGTGTTCCACTTCAAT |
| 28 | CaLTP39Rev | TGATGGAGTGGCTGTCTCAG |
| 29 | CaLTP40F | ATCACCCTGTCGCCTTGTCT |
| 30 | CaLTP40R | TGATGTTGATTCCCAGGGACG |
| 31 | CaLTP43For | CCACCATCTTCTTGCTGTGA |
| 32 | CaLTP43Rev | CTTGTTTGCAGGTGGTGAGA |
| 33 | CaLTP44For | GTTCCATGTCCTCCACCAC |
| 34 | CaLTP44Rev | GGTGTCTTTGGTGGCTTACC |
| 35 | CaLTP45For | CCCCTTCAAGTGGCTCATGT |
| 36 | CaLTP45Rev | GCTGCAGCCTGAAGATCAGT |
| 37 | CaLTP46For | GATTGGGTCACCACCAGAAC |
| 38 | CaLTP46Rev | GAGGGTGGAGTCTTCTCACA |
| 39 | CaLTP48For | GGACTGTGTCCTTGTTGTGG |
| 40 | CaLTP48Rev | GGTTGGCCCAAACCCTTA |

**Supplementary Table 7:** Prediction of possible miRNAs that can bind with the CaLTPs and can cause inhibition.

| miRNA_Accession. | Target_  Accession. | Expectation | UPE | miRNA_aligned_fragment | Target_aligned_fragment | Inhibition |
| --- | --- | --- | --- | --- | --- | --- |
| bdi-miR7710-3p | CaLTP18 | 2 | 19.377 | AUUGAUGUCACAAACUAUAGUAGC | AUAGCUAUAGUUUUUGCCAUCAAU | Translation |
| hme-miR-6309-3p | CaLTP21 | 2 | 14.945 | AUGGUCUUCAUUUUCUACUUCG | AAGAGU-GAAGAUGAAGACCAU | Cleavage |
| ppt-miR1222b | CaLTP15 | 2 | 16.379 | CUGAAGGAGUUCAUUGGUACA | UGUACCAAUGGACUUCUUUGG | Cleavage |
| ppt-miR1222b | CaLTP20 | 2 | 18.625 | CUGAAGGAGUUCAUUGGUACA | UGUACCAAUGGACUUCUUUGG | Cleavage |
| ppt-miR1222c | CaLTP15 | 2 | 16.379 | CUGAAGGAGUUCAUUGGUACA | UGUACCAAUGGACUUCUUUGG | Cleavage |
| ppt-miR1222c | CaLTP20 | 2 | 18.625 | CUGAAGGAGUUCAUUGGUACA | UGUACCAAUGGACUUCUUUGG | Cleavage |
| tae-miR530 | CaLTP23 | 2 | 15.535 | UGCAGUGGCAUAUGCAACUCU | AGAGUUGCAU--GCCACUGCA | Translation |
| bdi-miR7713-3p | CaLTP13 | 2.5 | 12.819 | UUGAGACUGGCAGCAUUAGCAAGC | UUCGUCUAUUGCUGCUAGUCUCAA | Cleavage |
| bdi-miR7755-5p | CaLTP38 | 2.5 | 20.025 | AUUCCACACUGUAAAUUGAAU | CUUCAAUG-AUAGUGUGGAAU | Cleavage |
| csi-miR3948 | CaLTP24 | 2.5 | 11.574 | UGGAGUGGGAGUGGGAGUAGGGUG | UGUCCUACUCCAGCUUCCGCUCCA | Cleavage |
| csi-miR3948 | CaLTP25 | 2.5 | 10.52 | UGGAGUGGGAGUGGGAGUAGGGUG | UGUCCUACUCCGGCUUCCGCUCCA | Cleavage |
| ddi-miR-7099 | CaLTP29 | 2.5 | 15.394 | UAAUCCUCAUUUAUUGCAUUA | CAAUGCAA-AGAAGAGGAUUA | Translation |
| gra-miR530a | CaLTP28 | 2.5 | 9.23 | AGGUGCAGAUGCAGUUGCAGG | CCUUCAACUCCAUCUGCAUCU | Cleavage |
| mtr-miR2660 | CaLTP22 | 2.5 | 15.028 | UAAGACAUCAGCUAUAAGCUA | GAGUUUGU-GCUGAUGUUUUA | Cleavage |
| pde-miR3704a | CaLTP32 | 2.5 | 20.241 | GGUCUCGGUGGAGUUGGGAAGA | UAUUCCCAAUUUCACUGGGAUC | Cleavage |
| ptc-miR169y | CaLTP8 | 2.5 | 20.303 | UAGCCAUGGAUG-AAUUGCCUG | AAGGUAAUUACAUGCAUGGCUA | Translation |
| aly-miR861-5p | CaLTP19 | 3 | 15.583 | GUUUGGAGAAAUAUGCAUCAU | CUGAUG-AUAUUUCUCUGAAA | Cleavage |
| ata-miR408-5p | CaLTP14 | 3 | 15.75 | CAGGGAUGGAGCAGAGCAAGG | UCUUGCUCU-CUCUCUCCCUG | Cleavage |
| ath-miR5645a | CaLTP35 | 3 | 16.552 | AUUUGAGUCAUGUCGUUAAG | CUUAAUGCCAUGGCUCAAAC | Cleavage |
| ath-miR5645b | CaLTP35 | 3 | 16.552 | AUUUGAGUCAUGUCGUUAAG | CUUAAUGCCAUGGCUCAAAC | Cleavage |
| ath-miR5645d | CaLTP35 | 3 | 16.552 | AUUUGAGUCAUGUCGUUAAG | CUUAAUGCCAUGGCUCAAAC | Cleavage |
| ath-miR5645e | CaLTP35 | 3 | 16.552 | AUUUGAGUCAUGUCGUUAAG | CUUAAUGCCAUGGCUCAAAC | Cleavage |
| ath-miR5645f | CaLTP35 | 3 | 16.552 | AUUUGAGUCAUGUCGUUAAG | CUUAAUGCCAUGGCUCAAAC | Cleavage |
| ath-miR8168 | CaLTP8 | 3 | 24.724 | AGGUGCUGAGUGUGCUAGUGC | CCACUA-CAGAGUCAGCACCU | Translation |
| bdi-miR5177 | CaLTP35 | 3 | 18.11 | UGAGGUUGUAAAACAGACAGU | CUUGUCUGUUUUGCAAUUUUG | Cleavage |
| bdi-miR7729a-5p | CaLTP19 | 3 | 16.649 | UGUUUUCA-UAGGCCAUGUAGAGC | AAGCUACAUGGACUAGUGGAAGCA | Cleavage |
| bdi-miR7729b-5p | CaLTP19 | 3 | 16.649 | UGUUUUCA-UAGGCCAUGUAGAGC | AAGCUACAUGGACUAGUGGAAGCA | Cleavage |
| ghr-miR2949b | CaLTP38 | 3 | 20.407 | UCUUUUGAACUGGAUUUGCCGA | AUGGCAGAUUCA--UCAAAAGA | Translation |
| ghr-miR2949c | CaLTP38 | 3 | 20.407 | UCUUUUGAACUGGAUUUGCCGA | AUGGCAGAUUCA--UCAAAAGA | Translation |
| gma-miR4356 | CaLTP11 | 3 | 16.838 | CAGGACUGUCUUAGAAAGCCAGGC | CAUUGGUUAUCUAAGG-AGUCCUG | Cleavage |
| gma-miR9757 | CaLTP32 | 3 | 20.119 | CAACCCUCCUCAGUUAGAUCUC | UUGAUCUAGCUGAAGGGGGUUC | Translation |
| gra-miR8633 | CaLTP45 | 3 | 6.56 | UAAGUGAAGA-AAGAGGUAGGUU | AACCU-CCUCUUCUUUUCACUUG | Translation |
| hvu-miR6184 | CaLTP39 | 3 | 20.668 | CGGCGUCG-GAUCUGGCCGGCCU | CCUACGGCCAGAUCACGACGCCG | Cleavage |
| mtr-miR2660 | CaLTP25 | 3 | 18.45 | UAAGACAUCAGCUAUAAGCUA | GGGUUUGU-GCUGAUGUUUUA | Cleavage |
| mtr-miR5237 | CaLTP4 | 3 | 18.169 | UUCAAAAGAUUUAGUUGGGAU | AUUCCAG--AAAUCUUUUGAA | Cleavage |
| mtr-miR5298b | CaLTP16 | 3 | 13.368 | UGAUGGAGA-UGA-UAUGAAGAUGAA | GUUUUCUUCAUGCUCAAUCUUCAUCA | Translation |
| mtr-miR5298c | CaLTP16 | 3 | 13.368 | UGAUGGAGA-UGA-UAUGAAGAUGAA | GUUUUCUUCAUGCUCAAUCUUCAUCA | Translation |
| osa-miR1439 | CaLTP29 | 3 | 13.609 | UUUUGGAACGGAGUGAGUAUU | GAUAUUCAUUCUAUUCUAAAA | Translation |
| osa-miR1861d | CaLTP39 | 3 | 19.329 | UGGUCUUGAGGCAGGAACUGAG | ACCACUUCCUGCUCCAGGACCA | Translation |
| osa-miR5518 | CaLTP16 | 3 | 22.93 | AUACUCAAACAGGGCAUUGCA | UGCAAUGUCUGGGUUGAGUAU | Translation |
| ppt-miR1051-5p | CaLTP44 | 3 | 16.945 | GGUUCAAGUGAACAGGAAGA | UCUUCCUAUUAACUUGAGCU | Translation |
| ppt-miR1222a | CaLTP15 | 3 | 16.379 | UUGAAGGAGUUCAUUGGUAUA | UGUACCAAUGGACUUCUUUGG | Cleavage |
| ppt-miR1222a | CaLTP20 | 3 | 18.625 | UUGAAGGAGUUCAUUGGUAUA | UGUACCAAUGGACUUCUUUGG | Cleavage |
| ptc-miR6425a-5p | CaLTP48 | 3 | 20.562 | UUGUCUUCCAUGGAAUAGGCAG | CAGCUUAUUCCAUGUAGGGCAG | Cleavage |
| ptc-miR6425b-5p | CaLTP48 | 3 | 20.562 | UUGUCUUCCAUGGAAUAGGCAG | CAGCUUAUUCCAUGUAGGGCAG | Cleavage |
| ptc-miR6425c-5p | CaLTP48 | 3 | 20.562 | UUGUCUUCCAUGGAAUAGGCAG | CAGCUUAUUCCAUGUAGGGCAG | Cleavage |
| ptc-miR6425d-5p | CaLTP48 | 3 | 20.562 | UUGUCUUCCAUGGAAUAGGCAG | CAGCUUAUUCCAUGUAGGGCAG | Cleavage |
| ptc-miR6425e | CaLTP48 | 3 | 20.562 | UUGUCUUCCAUGGAAUAGGCAG | CAGCUUAUUCCAUGUAGGGCAG | Cleavage |
| rgl-miR7797b | CaLTP21 | 3 | 7.164 | UUUGAUUUCGUCUUACAUUUUUC | GAGAAAUG-GAGAAGAAAUCAGA | Translation |
| stu-miR7979 | CaLTP10 | 3 | 15.289 | AGGUACAUGAACUCUAACGAGGCA | ACUCUUGUUA-AGUUUGUGUGCCU | Cleavage |
| zma-miR167c-3p | CaLTP13 | 3 | 17.452 | GAUCAUGCUGUGGCAGCCUCACU | UGAGAUGCUGCUA-AGCAUGGUC | Translation |
